# Development of novel monoclonal antibodies against starch and ulvan - implications for antibody production against polysaccharides with limited immunogenicity

**DOI:** 10.1038/s41598-017-04307-2

**Published:** 2017-08-24

**Authors:** Maja G. Rydahl, Stjepan K. Kračun, Jonatan U. Fangel, Gurvan Michel, Alexia Guillouzo, Sabine Génicot, Jozef Mravec, Jesper Harholt, Casper Wilkens, Mohammed Saddik Motawia, Birte Svensson, Olivier Tranquet, Marie-Christine Ralet, Bodil Jørgensen, David S. Domozych, William G. T. Willats

**Affiliations:** 1 Department of Plant and Environmental Sciences, DK-1871 Frederiksberg, Denmark; 2 Biology Department, Skidmore College, Saratoga Springs, NY, 12866 USA; 3 Carlsberg Research Laboratory, J.C. Jacobsens Gade 4, DK-1799 Copenhagen V, Denmark; 4 Sorbonne Universités, UPMC Univ Paris 06, CNRS, UMR 8227, Integrative Biology of Marine Models, Station Biologique de Roscoff, CS 90074 Roscoff, Bretagne France; 5 Department of Biotechnology and Biomedicine, Technical University of Denmark, DK-2800 Lyngby, Denmark; 6 UR1268 Biopolymeres, Interactions et Assemblages, Institut National de la Recherche Agronomique, Rue de la Géraudière, BP 71627, F–44316 Nantes, France; 7 School of Agriculture, Food and Rural Development, Newcastle University, NE1 7RU Newcastle upon Tyne, UK

## Abstract

Monoclonal antibodies (mAbs) are widely used and powerful research tools, but the generation of mAbs against glycan epitopes is generally more problematic than against proteins. This is especially significant for research on polysaccharide-rich land plants and algae (Viridiplantae). Most antibody production is based on using single antigens, however, there are significant gaps in the current repertoire of mAbs against some glycan targets with low immunogenicity. We approached mAb production in a different way and immunised with a complex mixture of polysaccharides. The multiplexed screening capability of carbohydrate microarrays was then exploited to deconvolute the specificities of individual mAbs. Using this strategy, we generated a set of novel mAbs, including one against starch (INCh1) and one against ulvan (INCh2). These polysaccharides are important storage and structural polymers respectively, but both are generally considered as having limited immunogenicity. INCh1 and INCh2 therefore represent important new molecular probes for Viridiplantae research. Moreover, since the α-(1-4)-glucan epitope recognised by INCh1 is also a component of glycogen, this mAb can also be used in mammalian systems. We describe the detailed characterisation of INCh1 and INCh2, and discuss the potential of a non-directed mass-screening approach for mAb production against some glycan targets.

## Introduction

Monoclonal antibodies (mAbs) are amongst the most widely used and powerful tools available to biology and medicine and their production exploits the inherent capacity of mammalian immune systems to discriminate self from non-self^1–6^. To produce mAbs, animals are immunised and antibody-producing B cells are subsequently removed and fused with myeloma cells. The resulting hybridoma cell lines are both immortal and antibody secreting. However, the recruitment of immune systems in live animals does have its limitations and relies on an antigen triggering the adaptive immune response. This is determined by several factors including chemical composition, heterogeneity, size, susceptibility to antigen processing, presentation and solubility. Although typically large, polysaccharides are generally far less immunogenic than proteins because whereas proteins generally display high internal molecular complexity and heterogeneity, polysaccharides are often constructed of regular repeating units and carbohydrates normally need to be conjugated to protein carriers to elicit an immune response^7–13^. Partly because of this, there are significant gaps in the current repertoire of polysaccharide-directed mAbs. This is particularly significant for research on plants and algae (collectively known as Viridiplantae), in which polysaccharides are the major constituents of their cell walls^14^. Our aim was to exploit advances in high-throughput (HTP) screening technology to facilitate the development of mAbs with novel specificities against Viridiplantae polysaccharides. We focused on Charophyta and Chlorophyta algae, chosen for their evolutionary significance and emerging biotechnological importance. Whilst the Chlorophyta gave rise to a large diversity of marine and freshwater green algae, the Charophyta gave rise to freshwater green algae and eventually to land plants^15^. Research into extant Charophyte species and their polysaccharides is critical for understanding the early evolution of land plants and their subsequent global radiation and ecological dominance. More molecular probes for Chlorophyte glycan research are required to better understand marine ecosystems and to harness the potential of algal biomass for bio-medical and industrial applications.

Most antibody production is based on immunising with a well-defined antigen in order to direct the immunological outcome. But some glycan targets have proven difficult or impossible to raise antibodies against - for example the ubiquitous plant cell wall polymers rhamnogalacturonan II, cellulose, starch and sulphated algal polysaccharides. Our approach was to immunise with an immunogen composed of a very complex mixture of polysaccharides (‘shotgun immunisation’) using a short immunisation regime, with high booster frequency with the aim of producing antibodies with diverse specificities. We then retrospectively deconvoluted their individual binding profiles using carbohydrate microarrays^16–18^. Using this strategy, we generated novel mAbs with specificity against starch and ulvan, two biologically and industrially significant polysaccharides with limited immunogenicity. To our knowledge, there are no anti-ulvan mAbs currently available and immune responses to starch are inhibited by the structural and compositional similarity of starch to the mammalian storage polysaccharide glycogen^19^. The fact that shotgun immunisation with a complex antigen mixture yielded two mAbs against these polysaccharides raises interesting questions about the underlying immunological events at play.

## Results and Discussion

### Immunogen selection and preparation

The immunogen used was prepared from 42 distinct Charophyte and Chlorophyte algae species (see Table S2), selected to cover a wide phylogenetic range.

It is clear from previous antibody production work that some Viridiplantae polysaccharides, notably some pectins, and proteoglycans, tend to be immunodominant. Therefore a disproportionally high number of mAbs against these glycans tends to emerge compared to other structures^20^. To counter this, we produced two fractions from our crude algal homogenate. One fraction was extracted using CDTA and was expected to contain the majority of pectic polymers and proteoglycans. A second fraction was extracted using NaOH and this was expected to contain polysaccharides held more tightly within cell wall architectures. The CDTA and NaOH fractions were used separately in different animals in hope that the potential immunodominance of pectin and proteoglycans would only affect animals immunised with the CTDA fraction.

Following hybridoma production, a pre-selection of mAb producing lines was carried out using standard enzyme-linked immunosorbent assays, in which plates were coated with the CDTA and NaOH immunogens. From this pre-screen, 27 mAbs, designated INCh1-27 (‘I**N**RA and Copenhagen’) with high binding were selected for further detailed characterisation.

### Initial microarray screening: plant and algae materials

Our aim was to identify mAbs that had novel binding profiles and first we screened them using microarrays populated with polysaccharides extracted with CDTA or NaOH from a variety of plants, and from the Charophyte and Chlorophyte algae used as immunogens. For comparison, and to validate the microarrays, we also included a selection of previously well characterised control mAbs with specificity for plant cell wall polysaccharides. The binding of all 27 INCh series mAbs, and previously characterised mAbs used as control to individual species of Viridiplantae are shown in Supplemental Figs S1–S3 respectively. As expected, the previously characterised plant directed mAbs bound to polysaccharides present in both plants and algae (Figs S1–S3). This is consistent with the fact that many plant polysaccharides first emerged in algae and then persisted during colonisation of land^21, 22^. However, most (21) of the INCh mAbs bound exclusively to material from algae with only few (6) binding to land plants.

The heatmaps in Supplemental Figs S1–S3 reveal individual mAb binding profiles. The strong binding of the previously characterised land plant directed mAbs to a wide variety of plant species is evident in Fig. S1, but some of the INCh mAbs also showed highly selective and in some cases, species-specific binding to plants. For example, INCh8 and INCh27 bound only to CDTA and NaOH extracted material from the lycophyte *Huperzia phlegmaria*, whilst INCh10 and INCh11 bound only to the angiosperm (flowering plant) species *Cornus mas*, *Arabidopsis thaliana* and *Viscum album*. Next, we examined mAb binding to microarrays populated with CDTA (Fig. S2) and NaOH (Fig. S3) extracted polysaccharides from the individual algae species that were collectively used as the immunogens. Some of the INCh mAbs bound to material from multiple species (for example, INCh7, INCh8 and INCh9) whilst some were highly species and/or extraction type specific. For example, INCh4, INCh5, and INCh6 bound only to the CDTA fraction from *Chlorella sp*. (Fig. S2) whilst INCh2 bound only CDTA and NaOH material from the two very closely related Ulvophyceae species *Ulva lactuta* (*U*. *lactuta*) and *Enteromorpha intestinalis* (*E*. *intestinalis*) (Figs S2 and S3).

Two extremes in terms of specificity were exemplified by the binding data for INCh1 (IgMκ) and INCh2 (IgG1λ) against NaOH extracted material (Fig. S3). Whilst INCh1 bound to almost all species, INCh2 bound only to material extracted from two Ulvophyceae species. The widespread binding of INCh1 raised the question of whether this mAb had broad specificity to multiple molecular structures, or more likely, bound specifically to target molecule that was present in a very wide range of organisms. INCh2 binding was significant because Ulvophyceae are globally distributed green seaweeds and are of considerable biotechnological importance as they represent a significant portion of macroalgal biomass. One of the most abundant non-cellulosic polysaccharide in the Ulvophyceae class is ulvan, a complex sulphated glucuronorhamnoxyloglucan against which there are no antibody probes currently available. INCh1 and INCh2 were selected for further detailed epitope characterisation.

### Defined polysaccharide screening

**Figure 1:**
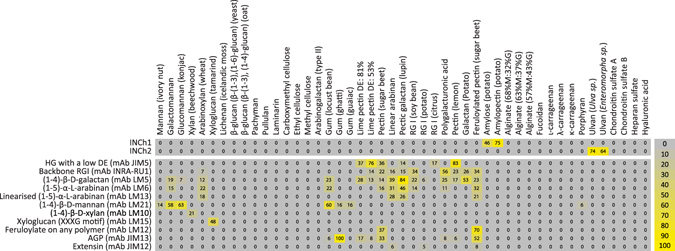
Characterisation of INCh1 and INCh2 using a microarray populated with diverse polysaccharides. Heatmap showing analysis of INCh1 and INCh2 binding using carbohydrate microarrays. Arrays where populated with polysaccharides from land plant, algal and animal sources. Note that INCh1 bound exclusively to starch and INCh2 to ulvan. Arrays were also probed with a set of previously characterised control mAbs. Colour intensity in the heatmap is correlated to mean spot signals. The highest signal in each dataset was set to 100, and all other values were normalized accordingly as indicated by the colour scale bar. A low-end cut-off value of 5 was imposed. Homogalacturonan (HG), arabinogalactan protein (AGP), rhamnogalacturonan (RG).

The epitopes recognised by INCh1 and INCh2 were explored using microarrays populated with 121 isolated and/or chemically synthesised oligo- and polysaccharides (see Fig. 1 and Table S1 for other ligands that did not elicit binding). The range of samples represented the major polysaccharide classes found in plants including pectins, proteoglycans and hemicellulosic polysaccharides and each polymer class was represented by multiple sample structures in most cases. A range of starch samples was also included, as well as control polymers that were not of plant or algal origin. These arrays were probed with INCh1 and INCh2 together with a set of previously characterised control mAbs (Fig. 1). The binding profiles obtained for INCh1 and INCh2 indicated that these mAbs bind to starch and ulvan respectively.

INCh1 bound to amylose and amylopectin, but not to any other samples on the array. The amylose component of starch is composed of linear α-(1-4)-linked glucose residues, whilst amylopectin has a branched structure where α-(1-4)-linked glucan chains are attached to each an α-(1-4) glucan backbone via α-(1-6)-linkages. With these structural features in mind, the INCh1 binding profile indicated that at least some linear α-(1-4)-linked glucan domains are required for binding, but that the presence of α-(1-6)-linkages did not preclude binding. The specificity of INCh1 towards starch is consistent with its widespread binding to diverse species (Figs S1–S3) since starch is a ubiquitous storage carbohydrate. INCh2 only bound to ulvan samples derived from two Ulvophyceae species - *Ulva sp*. and *Enteromorpha sp*. (Fig. 1). Again, this is consistent with the restricted binding of INCh2 seen in Fig. S3 because ulvan is a polysaccharide that is restricted to the Ulvophyceae class.

The specificities of INCh1 and INCh2 were investigated further using microarrays populated with oligosaccharides, polysaccharides, enzymatic epitope deletion, microscopy and in the case of INCh2, additional materials extracted from algae as well as monosaccharide composition analysis.

### Detailed epitope mapping

#### INCh1 epitope characterization

**Figure 2:**
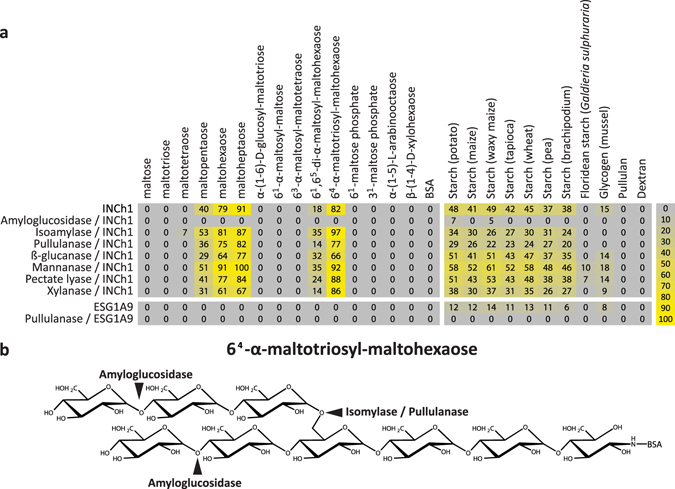
INCh1 characterisation using a microarray populated with starch-related samples. (**a**) Heatmap showing INCh1 binding to a carbohydrate microarray populated with starch-related oligo- and polysaccharides and enzymatic epitope deletion. Note that INCh1 bound to α-(1-4)-glucan oligomers with a minimum degree of polymerisation of 4. INCh1 binding was abolished by pre-treatment of arrays with amyloglucosidase but not by isoamylase, pullulanase or any non-starch related control enzymes. Colour intensity in the heatmap is correlated to mean spot signals. The highest signal in each dataset was set to 100, and all other values were normalized accordingly as indicated by the colour scale bar. A low-end cut-off value of 5 was imposed. (**b**) Diagram showing the cleavage sites of the starch degrading enzymes used.

Data from the first two screening levels strongly indicated that INCh1 is a starch-binding mAb and consequently we sought to define the epitope structure recognised. To do this, we screened INCh1 against a set of oligosaccharides representing common sub-structures found in starch as well as additional starch polysaccharide samples (Fig. 2). In addition, we investigated the effects of starch-degrading enzymes on INCh1 binding to these samples (Fig. 2a). To aid the interpretation of heatmap data, we have included chemical structures of the starch related oligosaccharides used (Fig. S4).

INCh1 bound to maltooligosaccharides but only when at least 4 contiguous α-(1-4)-linked glucose residues were present. Note that the covalent coupling of the oligosaccharides to the microarray surface entailed ring-opening of the reducing-end sugar residue, so that the maltopentaose sample had 4 and not 5 contiguous α-(1-4)-linked glucose residues and the maltotetraose sample only 3, *et cetera*. The degree of binding to maltooligosaccharides increased with the degree of polymerization (DP), so that the INCh1 binding was stronger towards maltoheptaose than towards maltohexaose and maltopentaose. INCh1 did not bind to α-(1-6)-D-glucosyl-maltotriose, 6^1^-α-maltosyl-maltose or 6^3^-α-maltosyl-maltotetraose, and this is consistent with a minimum requirement for 4 contiguous α-(1-4)-linked glucose residues for INCh1 binding. Since INCh1 bound to amylopectin on the polysaccharide arrays (Fig. 1), we also investigated INCh1 binding to oligosaccharides representing branched amylopectin sub-structures. INCh1 bound to both 6^1^, 6^5^-di-α-maltosyl-maltohexaose and to 6^4^-α-maltosyl-maltohexaose, with binding to 6^4^-α-maltosyl-maltohexaose considerably stronger than to 6^1^, 6^5^-di–α-maltosyl-maltohexaose. One explanation for this is that the presence of two branch points on 6^1^, 6^5^-di-α-maltosyl-maltohexaose interferes with INCh1 binding to α-(1-4)-linked glucan more than the single branch point on 6^4^-α-maltosyl-maltohexaose. In addition to the oligosaccharides, INCh1 binding to starch derived from a variety of plant species was also assessed (Fig. 2a). These data indicated that INCh1 bound to starch from diverse species, but not to floridean starch (a storage polysaccharide of red algae). Most starch from Viridiplantae contains both amylose and amylopectin domains, but floridean starch lacks amylose^23^, supporting the notion that the INCh1 epitope comprises α-(1-4)-linked glucose residues.

A further level of characterisation was provided by digesting the samples on the arrays with starch-degrading enzymes (as well as control enzymes) prior to INCh1 probing (Fig. 2a). The schematic in Fig. 2b shows the cleavage sites of the starch degrading enzymes used. INCh1 binding was abolished by amyloglucosidase pre-treatment, providing further evidence that the INCh1 epitope contains α-(1-4)-linked glucan. In contrast, isoamylase and pullulanase did not appreciably affect INCh1 binding, indicating that α-(1-6)-linked glucose residues are not required for INCh1 binding. Non-starch related control enzymes did not significantly affect INCh1 binding (Fig. 2a).

As discussed, α-(1-4)-linked glucan is a component of amylose and amylopectin, but it is also a major structural feature of the animal storage polysaccharide glycogen and we investigated INCh1 binding to glycogen on arrays (Fig. 2a). Binding to glycogen was notably weaker than to starch, consistent with the fact that glycogen is heavily branched with α-(1-6)-linkages and contains proportionally less α-(1-4)-linked glucan^24^. To investigate this further, INCh1 binding was compared to the glycogen-directed mAb ESG1A9 (Fig. 2a), which recognises highly branched glycogen and, with much lower affinity, also starch^25^. On our arrays, ESG1A9 bound weakly to diverse starch types of plants as well as glycogen, but not to any of the branched or unbranched oligosaccharides. Pre-treatment with pullulanase, a debranching enzyme that by cleaves α-(1-6)-linked glucose, abolished ESG1A9 binding, confirming that the epitope recognised by ESG1A9 includes the branch point of glycogen, and is distinct from that recognised by INCh1.

We further characterised INCh1 binding by using surface plasmon resonance (SPR) to assess the affinity of the epitope-paratope interaction. INCh1 (1418 response units (RU)) was immobilised on a streptavidin chip and gave an R_max_ of 3.2, which resulted in a stoichiometry of 2.5 (see Eq. 2, Materials and methods). RU from SPR sensorgrams (Fig. S5b) fitted to the steady state equation (Eq. 1, Materials and methods) assuming binding at one binding sites does not affect the other binding sites (Fig. S5a). Maltopentaose was used for binding, since it previously showed high binding on defined arrays, but biotinylation can affect INCh1 orientation on the chip effectively restricting access to maltopentaose binding sites so the stoichiometry was rounded up^26–28^ which implies at least three binding sites on INCh1. The *K*_D_ of 192 ± 17 µM (see Eq. 1, Materials and methods), indicated relatively high affinity compared to maltooligosaccharide binding enzymes often yielding *K*_D_ in the mM range^27–32^.

#### Cellular localisation of α-(1-4)-linked glucan using INCh1

**Figure 3:**
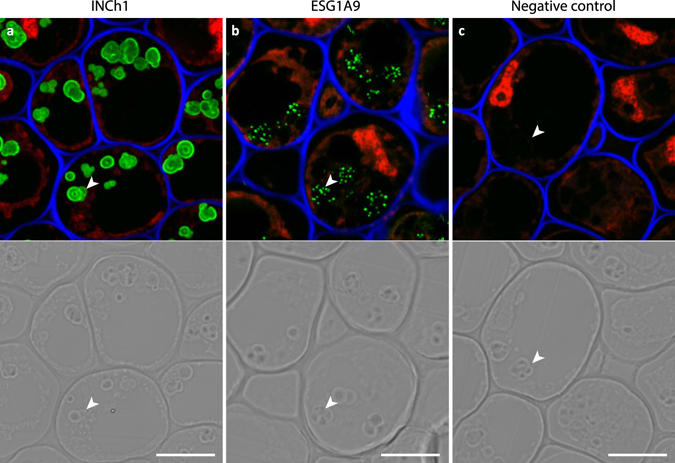
INCh1 and ECG1A9 mAb labelling of starch granules in pea root cap. Images showing immunofluorescence labelling of starch granules by INCh1 (**a**) and ECG1A9 (**b**) in resin-imbedded sections through pea root caps. (**c**) No primary antibody control. Images shown in (**a**–**c**) are overlays of signals from three channels: Calcofluor White (staining cellulose, blue), propidium iodide (staining nuclei, red) and immunolabeling using an anti-mouse conjugated Alexa Fluor 488 as secondary antibody (green). The lower panels show differential interference contrast images obtained from the same sections. Starch granules are marked with arrowheads. Scale bars = 10 μm.

**Figure 4:**
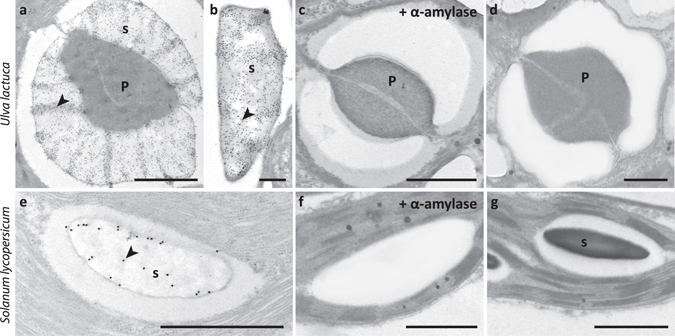
Immunogold transmission electron microscopy showing INCh1 binding to sections of *U. lactuca and Solanum Lycopersicum*. (**a**) Image showing INCh1 labelling of starch granules (s) surrounding the protein core of a pyrenoid (P) of the alga *U*. *lactuca*. (**b**) High magnification image showing INCh1 labelling of a single *U*. *lactuca* starch granule. (**c**) Image showing loss of INCh1 binding to *U*. *lactuca* starch granule when the section was treated with α-amylase prior to labelling. (**d**) Image from control labelling during which INCh1 was omitted in the labelling protocol. (**e**) Image showing immunogold labelling using INCh1 of a starch granule in a *S*. *lycopersicum* leaf. (**f**) Image showing loss of INCh1 binding to *S*. *lycopersicum* when the section was treated with α-amylase prior to labelling. (**g**) Image from control labelling during which INCh1 was omitted in the labelling protocol. Arrowheads indicate sites of gold particle labelling. Scale bars (**a**,**c**,**d**,**e**,**f**,**g**) = 1 μm; (**b**) = 200 nm.

INCh1 was used to as a probe to detect α-(1-4)-linked glucan in a variety of plant and animal systems, and in some cases binding was compared to that of ESG1A9. Labelling of thin resin-embedded sections through pea root caps with revealed that the INCh1 epitope was restricted to starch granules, with labelling being most intense at the surface of the granules (Fig. 3a). In contrast, when ESG1A9 was used to probe the same material, although the epitope was also restricted to starch granules, the labelling pattern was punctate (Fig. 3b). These differences in labelling attest to the distinct epitopes of INCh1 and ESG1A9, and suggest that plant starch granules contain glycogen-like subdomains. We also examined starch labelling by INCh1 in a variety of land plant and algal species using immunogold transmission electron microscopy (Fig. 4 and S6). In all cases, INCh1 labelling was restricted to starch granules and, in the case of *U*. *lactuca* and *Solanum lycopersicum* (tomato), labelling was abolished when sections were pre-treated with the α-(1-4)-linked glucan degrading enzyme α-amylase (Fig. 4).

Considering the presence of α-(1-4)-linked glucan in glycogen, and the binding of INCh1 to glycogen on arrays (Fig. 2), we were also interested to assess if INCh1 was an effective probe for α-(1-4)-linked glucan in animal systems. To do this, we applied INCh1 to paraffin sections of mouse skeletal muscles, and again also compared its binding to that of ESG1A9 (Fig. S7). Both antibodies revealed a granular overall labelling pattern but with subtly different cellular distributions across the muscle tissue. The labelling indicated that ESG1A9 binds the intermyofibrillar and subsarcolemmal glycogen, whereas INCh1 was somewhat more widespread and included the intramyofibrillar glycogen^33^ (Fig. S7).

Taken together, our data showed convincingly that INCh1 is a novel α-(1-4)-glucan specific mAb that requires a minimum of four contiguous glucose residues for binding. As discussed in relation to ESG1A9, glycogen-directed mAbs with the capability to also bind to starch have been described before. But to our knowledge, this is the first report of a mAb that preferentially binds to α-(1-4)-glucan with high specificity.

#### INCh2 epitope characterization

**Figure 5:**
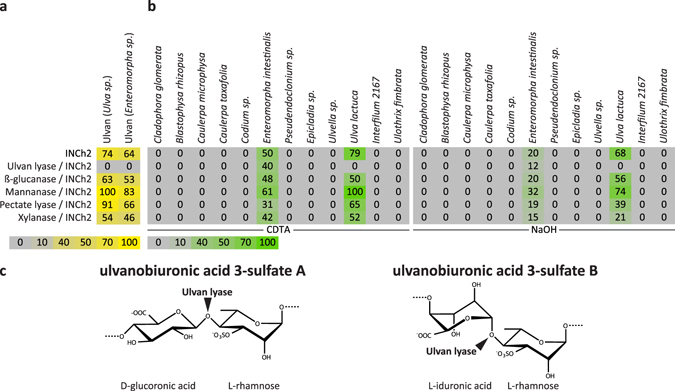
INCh2 characterisation using a microarray populated with ulvan polysaccharides and extracted cell wall material of diverse Ulvophycea species. (**a**) Heatmap showing INCh2 binding to a carbohydrate microarray populated with ulvan polysaccharides from the species *Ulva sp*. and *Enteromorpha sp*. and enzymatic epitope deletion. INCh2 binding was abolished by pre-treatment of arrays with ulvan lyase but not by any control enzymes. INCh2 binding was abolished by pre-treatment of arrays with ulvan lyase but not by control enzymes. (**b**) Heatmap showing INCh2 binding to a carbohydrate microarray populated with extracted cell wall material of diverse Ulvophycea species and enzymatic epitope deletion. The heatmap shows the relative abundance of cell wall components as extracted using CDTA and NaOH. The same amount of cell wall material (alcohol insoluble residue) was used for each sample. Note that INCh2 bound to *Ulva lactuca* and *Enteromorpha intestinalis* containing ulvan, but that INCh2 binding was only abolished by pre-treatment of arrays with ulvan lyase on *Ulva lactuca*. (**c**) Diagram showing the cleavage sites of the ulvan degrading enzyme used in the major disaccharide units found in ulvan. Colour intensity in the heatmap is correlated to mean spot signals. The highest signal in each dataset was set to 100, and all other values were normalized accordingly as indicated by the colour scale bar. A low-end cut-off value of 5 was imposed.

Initial array-based screening of INCh2 strongly indicated that this mAb is specific towards ulvan. To obtain further insight into the epitope recognised by INCh2, we used a set of arrays populated with ulvan samples from *Enteromorpha sp*. and *Ulva sp*. and crude polysaccharides mixtures extracted with CDTA and NaOH from an extended set of Ulvophyceae species (Fig. 5). Confirming the results from our initial screening INCh2 bound only to ulvan from *Enteromorpha sp*. and *Ulva sp*. (Fig. 5a), and only to polysaccharides extracted from the two Ulvophyceae species *E*. *intestinalis* and *U*. *lactuca* (Fig. 5b).

Ulvan is a complex sulphated polysaccharide, comprised of rhamnose-3-sulfate, xylose, xylose-2-sulfate, glucuronic acid and iduronic acid units^34, 35^. One prominent repeating disaccharide motif of ulvan was identified by Lahaye *et al*.^36^ as ulvanobiuronic acid 3-sulfate A, consisting of an α-1,4-linked repeating unit D-glucuronosyl-β-1,4-L-rhamnose-3-sulfate. The second major disaccharide motif is ulvanobiuronic acid 3-sulfate B, with the structure of α-1,4- linked repeating unit L-iduronosyl-α-1,4-L-rhamnose-3-sulfate^37^. These key structures are shown schematically in Fig. 5c. Considering the abundance of these disaccharides in ulvan, we speculated that

**Figure 6:**
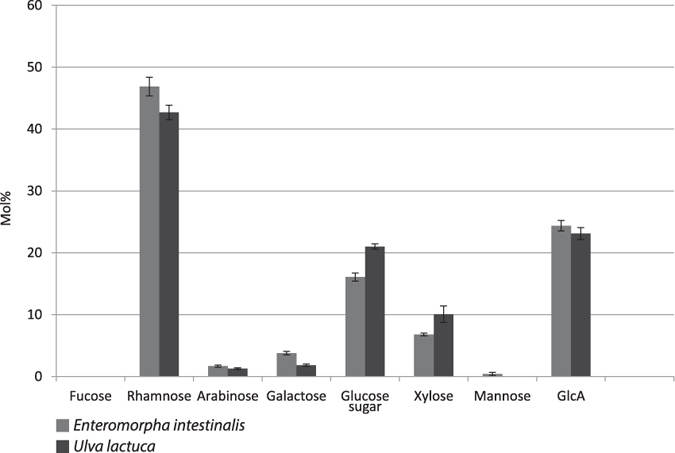
Sequential fractionation of *Ulva* lactuca and of *Enteromorpha* intestinalis analysed with quantitative sugar composition analysis. Monosaccharide composition (mol%) of *Ulva lactuca* and *Enteromorpha intestinalis* cell wall material. Note that differences in cell wall composition between *Ulva lactuca* and *Enteromorpha intestinalis* observed in the analysis of extracted cell wall material using INCh2 (Fig. 5), could also be detected here.

INCh2 was likely to bind to an epitope present in one of them. To investigate this, we used ulvan lyase as a tool for epitope deletion studies. The ulvan lyase sequence was originally identified in marine bacterium *Nonlabens ulvanivorans* PLR. This enzyme specifically cleaves the β-1,4-glycosidic bond between L-rhamnose-3-sulfate and the uronic acid and is therefore potentially a useful tool for ulvan epitope characterisation^38^. However, recombinant *N*. *ulvanivorans* ulvan lyase proved to be problematic to produce in useful quantities. The expressed protein could be detected by Western-blotting (Fig. S8a) using an anti-His antibody, but not in SDS-PAGE gels (Fig. S8b). We therefore identified a homologous ulvan lyase encoding gene (BN863_22190, Genbank: WP_038530530) in the genome of the marine bacterium *Formosa agariphila* ^39^ (*F*. *agariphila*). The corresponding protein is modular and includes an N-terminal signal peptide, a catalytic module, a putative carbohydrate module and a C-terminal type IX secretion module. This protein displays 52% identity with the entire sequence of the ulvan lyase from *N*. *ulvanivorans* and the catalytic modules of these two enzymes share 66% sequence identity. The recombinant expression in *E*. *coli* of the catalytic module of BN863_22190 (residues 25–285) yielded a substantial amount of soluble protein (~30 mg per litre of culture) (Fig. S8c,d). The ulvan lyase activity of BN863_22190 was confirmed by monitoring the release of unsaturated oligo-ulvans by UV spectrophotometry^40^. After extensive degradation of ulvan, the terminal products of this ulvan lyase were purified by size-exclusion chromatography and characterized by mass spectrometry (platform BIBS, INRA Nantes, France) as the disaccharide Δ-Rha3S and the tetrasaccharide Δ-Rha3S-Xyl-Rha3S. Therefore, BN863_22190 cleaves the β-1,4-glycosidic between L-rhamnose-3-sulfate and uronic acid residues and its products are similar to that of the wild-type ulvan lyase from *N*. *ulvanivorans* ^38, 40^. The cleavage site of BN863_22190 is shown in Fig. 5c. When this ulvan lyase was applied to arrays prior to labelling with INCh2, binding to ulvan from both *Enteromorpha sp*. and *Ulva sp*. was abolished, strongly indicating that the epitope recognised by INCh2 contains ulvanobiuronic acid 3-sulfate A and/or B (Fig. 5a). None of the control enzymes (glucanase, mannanase, pectate lyase and xylanase) had significant effects on INCh2 binding (Fig. 5a). Interestingly, when we analysed the effects of ulvan lyase treatment on INCh2 binding to polysaccharides extracted from *U*. *lactuca* and *E*. *intestinalis*, we found that binding to *U*. *lactuca* samples was abolished, but this was not the case for *E*. *intestinalis* (Fig. 5b). One likely explanation of this is that ulvan occurs in somewhat different macromolecular contexts in these two species, such that in crude *E*. *intestinalis* polysaccharide extractions, the ulvan lyase cleavage site is masked or sterically hindered by other cell wall components. In the purified ulvan samples this difference may be absent, allowing the enzyme to degrade the INCh2 epitope in ulvan from both species. The different effects of ulvan lyase on INCh2 binding to *U*. *lactuca* and *E*. *intestinalis* polysaccharide extractions is of interest because the phylogenetic positioning of these two species is disputed and it is not clear if they are taxonomically distinct or not refs 34, 41. Uncertainty about classification may be due to phenotypic changes that occur when the algae are grown under different growth conditions^34, 41^. Our data suggest that *U*. *lactuca* and *E*. *intestinalis* are in fact distinguishable on the basis of ulvan structure and we investigated this further by analysing the monosaccharide compositions of the two species (Fig. 6). This analysis revealed significant differences in the levels of several sugars including rhamnose, glucose, galactose, xylose and galacturonic acid.

#### Cellular localisation of ulvan using INCh2

**Figure 7:**
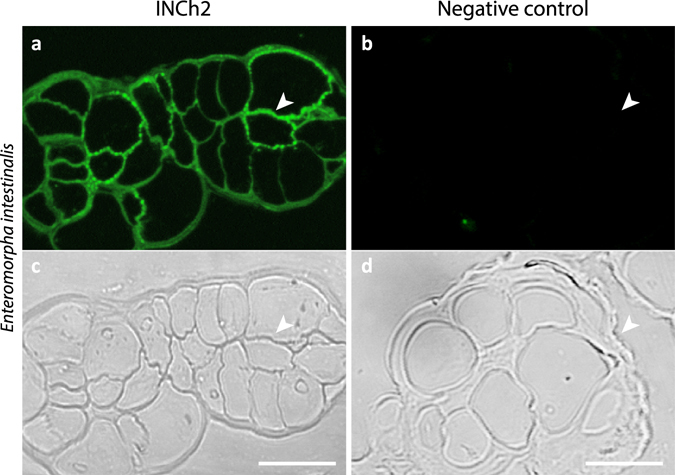
INCh2 mAb labelling cell wall in *Enteromorpha* intestinalis. (**a**) Images showing immunofluorescence labelling of cell wall by INCh2 using an anti-mouse conjugated Alexa Fluor 488. (**b**) No primary antibody control. The lower panels show differential interference contrast images obtained from the same sections. Cell walls are marked with arrowheads. Scale bars = 10 μm.

**Figure 8:**
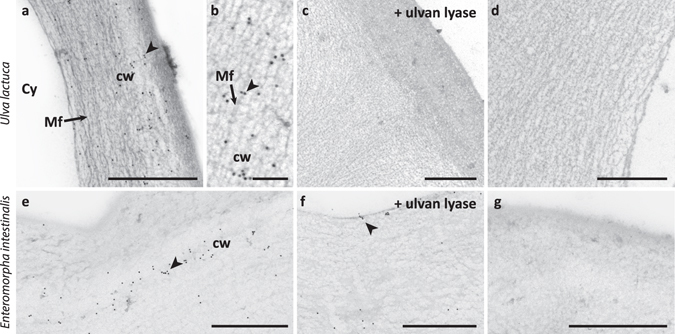
Immunogold transmission electron microscopy showing INCh2 binding to sections of *Ulva lactuca* and *Enteromorpha intestinalis*. (**a**) Image showing INCh2 labelling of cell wall (cw) of the alga *Ulva lactuca*. (**b**) High magnification image showing INCh2 labelling *Ulva lactuca* cell wall. (**c**) Image showing loss of INCh2 binding to *Ulva lactuca* cell wall when the section was treated with ulvan lyase prior to labelling. (**d**) Image from control labelling during which INCh2 was omitted in the labelling protocol. (**e**) Image showing immunogold labelling using INCh2 of the cell wall in the alga *Enteromorpha intestinalis*. (**f**) Image showing immunogold labelling using INCh2 when the section was treated with ulvan lyase prior to labelling. (**g**) Image from control labelling during which INCh2 was omitted in the labelling protocol. Note that INCh2 bound to *Ulva lactuca* and *Enteromorpha intestinalis* containing ulvan, but that INCh2 binding was only abolished by pre-treatment of sections with ulvan lyase on *Ulva lactuca*. Arrowheads indicate sites of gold particle labelling. Scale bars (**a**,**c**,**d**,**e**,**f**,**g**) = 1 μm; (**b**) = 200 nm. Cell wall (cw), cytosol (Cy) and microfibrillar (Mf).

Next, we explored the use of INCh2 as a probe for localising ulvan in cell walls or sectioned *U*. *lactuca* and *E*. *intestinalis* materials. Immunofluorescence labelling of *E*. *intestinalis* revelled cell wall localised labelling (Fig. 7). Immunogold TEM labelling clearly indicates that INCh2 labels the cell wall of both *U*. *lactuca* and *E*. *intestinalis* (Fig. 8a,b,e). It is worth noting that as with the polysaccharide extracts (Fig. 5b), pre-treatment of sections with ulvan lyase only removed the epitope from *U*. *lactuca* and not of *E*. *intestinalis* (Fig. 8c,f). Again, this indicates that there are differences between ulvan in *U*. *lactuca* and *E*. *intestinalis* at the macromolecular and/or cell wall architecture levels.

## Conclusions

The INCh1 and INCh2 mAbs represent significant additions to the glycan research toolbox and demonstrate the value carbohydrate microarray technology has for rapid specificity screening. This work may also provide some insight into how the barrier of limited immunogenicity may be overcome in some cases. The paucity of starch-specific antibodies is often assumed to be related to the similarity of starch to glycogen which triggers the innate capacity for discrimination of self *versus* non-self in mammalian immune systems^42^. However, it is known that in some cases, immune system overload can be triggered via post-thymic *de novo* T-cell receptor (TCR) editing^43^, resulting in activation of B-cells that normally would not be activated using traditional immunisation protocols^43^. Moreover, with the administration of early and frequent booster immunizations, the immune response is likely to produce a broader response in terms of specificity^44^. Together, the result of these effects may produce an immune response targeted towards normally non-antigenic targets while still maintaining self-tolerance and foreign-antigen reactivity - a characteristic of thymically selected TCRs^45, 46^ (see Fig. S9). Although this explanation is speculative, our work nonetheless raises some interesting considerations for future mAb production. Specifically, shotgun immunisation to produce diverse specificities, combined with the capability to de-convolute binding profiles of individual mAbs retrospectively using HTP screening technology, may be an approach that can be of value in relation to polysaccharides with low immunogenicity.

## Materials and Methods

### Biological materials

Algae where grown as previously described by Nichols^47^ in collaboration with The Skidmore College Algal Collection and The Scandinavian Culture Collection of Algae and Protozoa (see Table S2 for details on growth conditions and growth media). Cell wall polymers were isolated from cultures of diverse algae, as previously described Rydahl *et al*. 2014^48^. The pea seeds (*Pisum sativum* var. Norli) were sterilised with sodium hypochlorite, washed with sterile water 3 times and soaked in water for 3 hours and germinated in petri dishes with sterile water moistened paper in the dark at room temperature for 3 days.

### Immunogen production

The immunogen was produced by selecting 42 distinct charophytes and chlorophytes (see Table S2), carefully chosen to cover as wide and diverse and range of species as possible. The harvested mass of each alga was snap freezed with liquid nitrogen, and homogenized to a fine powder using a tissue lyser Qiagen MM 200 (Qiagen). The homogenate was incubated with 30 × volume of 50 mM 1,2- diaminocyclohexanetetraacetic acid (CDTA) pH 7.5 for 3 H at 18 °C. This was then centrifuged for 30 min at 4400 rpm, supernatant collected and stored in the fridge, and the pellet put for another extraction using CDTA for 16 H at 18 °C. This was then centrifuged for 30 min at 4,400 rpm*. The combined CDTA supernatants were collected and dialysed for 5 × 1,5 H using a Slide-A-Lyzer G-2 cassette system (Pierce) against deionised water (dH_2_O) (3,5 kDa molecular weight cut off) to remove low molecular weight molecules. The polysaccharides where precipitated using cold 96% EtOH (stored at −20 °C O/N). Polysaccharides where collected by centrifugation for 30 min at 4,400 rpm. Supernatant was removed and pellet was dried with acetone (labelled the ‘Pectic fraction’).

Meanwhile the pellet from $\ast$ was incubated with 30 × volume of 4 M NaOH with 1% m/v NaBH4, for the same time, dialysis and subsequent dried as with the CDTA extracts (labelled the ‘Hemicellulose fraction’).

The remaining pellet was washed five times with cold 96% EtOH and dried with acetone (labelled the ‘Pellet fraction’).

### Antibody generation

Three BALB/c mice were injected subcutaneously in the rear legs twice at two-week intervals with 20 µg of the Pectic fraction emulsified in Titermax Gold Adjuvant (Sigma). Three days after the second immunization, mice were sacrificed and the popliteal lymph nodes removed. Lymph nodes cells were suspended by perfusion and fused with a NS1 myeloma cell line using standard hybridoma preparation and limiting dilution cloning procedures. The hybridoma supernatants from the fusion were screened for the presence of antibodies reacting with the Pectic fraction by indirect ELISA. The selected hybridomas were subcloned by dilution. Crude hybridoma supernatants were used as the source of monoclonal antibodies. Animal experiments were carried out at the INRA facilities in Nantes, which are authorized by the Local Veterinary Department (authorization no. 44502). MAb production was approved by the Ethical Comity of Pays de la Loire under the number CEEA-2011.26. All methods were carried out in accordance with relevant guidelines and regulations.

After immunisation with the pectic fraction, we fused the immune cells and obtained 800 hybridomas. 50 of these hybridomas secreted antibodies specific for the Pectic fraction with no reactivity to the negative control (BSA). 10 hybridomas were selected for subclonning and liquid nitrogen preservation. On the other hand, immunization with the Hemicellulose fraction resulted in only 64 hybridomas.

### Indirect ELISA

For coating 100 µL of CDTA extract diluted at 10 µg/ml in carbonate buffer (50 mM, pH = 9.6) was applied to each well of a 96 well Maxisorb ELISA Plate (NUNC) and incubated overnight at +4 °C. Remaining binding sites were blocked with 4% defatted skimmed milk in phosphate-buffered saline (PBS) at pH 7.4 for 1 h. Hybridoma supernatants were then added and incubated for 1 h at room temperature. After washing with PBST (PBS with 0.05% Tween 20), bound antibodies were detected by incubation with horseradish peroxidase (HRP) tagged anti-mouse IgG (Bio-Rad, 1:3000 dilution) and *ortho*-phenylenediamine as substrate. Prior to detection with the tagged anti-mouse IgG, the secondary antibody was assessed to cross react with IgM antibodies to a satisfactory level (see supplementary information Table S3). Colour development was stopped with 100 µl of 2 M H_2_SO_4_ and the absorbance was read at 492 nm.

The Hemicellulose fraction could not be immobilised using the ELISA setup, hence this fraction was printed as carbohydrate microarrays. Among the Hemicellulose hybridomas, 28 were positive binders on the immunogen carbohydrate microarrays. Only 17 of these hybridomas survived to the subclonning and freezing steps and exhibited no reactivity to a negative control (BSA).

Isotype determination of the selected hybridomas was carried out using the Rapid ELISA Mouse mAb Isotyping Kit from Pierce, Thermo Fisher (Cat. No. 37503) with undiluted crude cell culture supernatant.

### SDS-PAGE and western blot of INCh1 and INCh2

INCh1 and INCh2 culture supernatants were partially purified by ultracentrifugation (50 kDa MWCO filters). 30 µl of supernatant was applied on 4–20% Stain Free Tris-HCI gels (Biorad, France) and separated by SDS-PAGE. Proteins were transferred onto 0.22 µm membrane (BioRad, France) using semi dry transfer (TransBlot Turbo, Biorad). After saturation with PBS containing 4% BSA (Sigma, France), the membrane was incubated with horseradish peroxidase conjugated anti mouse IgM (1/10,000, art. no. A8687, SIGMA) or horseradish peroxidase conjugated anti mouse IgG (1/10,000, art. 170-6516, BioRad) diluted in PBS containing 0.1% BSA. Detection of specific bands was performed using the ECL reagent (Western Bright Quantum, Advansta, USA) and cooled digital camera (LAS3000, Fuji) (see supplementary information Fig. S10). While INCh1 appears to be homogeneous, there is some heterogeneity displayed by INCh2. However, no indication of detected impurities affecting the specificity and binding studies has been found.

### Extraction of cell wall (alcohol insoluble residues, AIR)

AIR was prepared essentially as previously described by Fry^49^. To ensure the removal of hydrophobic compounds like lipids, a chloroform/MeOH (1:1 v/v) step was added to the procedure as demonstrated by Foster *et al*.^50^.

### Extracted carbohydrate microarrays (Comprehensive microarray polymer profiling (CoMPP))

CoMPP was performed as previously described in Moller *et al*.^16^, Fangel *et al*.^51^ and Kračun *et al*.^18^. In brief, cell wall polymers were sequentially extracted from 10 mg of AIR with 50 mM 1,2-diaminocyclohexanetetraacetic acid (CDTA), pH 7.5, followed by extraction with 4 M NaOH with 0.1% m/V NaBH_4_, and extractions printed in four dilutions and two replicates giving a total of 8 spots per sample. The same amount of cell wall material (AIR) was used for each sample.

### Poly- and oligosaccharide samples

Oligosaccharides and polysaccharides were purchased or donated by collaborators, see Table S1 for details.

### Carbohydrate microarrays printing, probing and analysis (with/without enzyme treatment)

Defined and extracted glycan microarrays were printed as described previously by Pedersen *et al*. and Moller *et al*.^16, 17^. For epitope removal on arrays using enzymes, the arrays were blocked with for 1 h in MPPBS, followed by three washes with PBS buffer. Arrays where then incubated with either PBS or commercially available enzymes in the appropriate buffer O/N at pH and temperature optima (see Table S4). After incubation, arrays where washed three times with PBS (each for 10 min), probed and quantified as previously described.

### Cloning, expression and purification of Ulvan ulvan lyases from marine bacteria

The genes encoding the ulvan lyase from *Nonlabens ulvanivorans* PLR and from *Formosa agariphila* DSM 15362 were cloned as in Groisillier *et al*.^52^. Briefly primers were designed to amplify the coding region corresponding to the catalytic module of the ulvan lyases by PCR from genomic DNA from *N*. *ulvanivorans* PLR (primer forward 5′ GGGGGGAGATCTCAAACAGCGCCTGATGAGGATAC 3′, primer reverse 5′ CCCCCCCAATTGTTAACTATTATCTACAACTTCTACTTTCTG 3′) and from *F*. *agariphila* DSM 15362 (primer forward 5′ GGGGGGGGATCCCAAACCGCGCCCGATGAAGATAC 3′; primer reverse 5′ CCCCCCCAATTGTTATGTAACCTCTACGTTTTCTACCTTTT 3′). After digestion with the restriction enzymes (BglII/MfeI for *N*. *ulvanivorans*; BamHI and MfeI for *F*. *agariphila*), the purified PCR products were ligated using the T4 DNA ligase into the expression vector pFO4 predigested by BamHI and EcoRI, resulting in recombinant proteins with a N-terminal hexa-histidine tag. The plasmids were transformed into Escherichia coli DH5α strain for storage and in *E*. *coli* BL21(DE3) strain for protein expression.

*E*. *coli* BL21(DE3) cells harbouring the ulvan lyase plasmids were cultivated at 20 °C in auto-induction ZYP 5052 medium (Studier, 2005, Protein Expr Purif) supplemented with 100 µg · mL^−1^ ampicillin (2 mL for protein expression screening; 1 L for protein production). Cultures were stopped when the cell growth reached the stationary phase and were centrifuged for 35 min at 4 °C, 3000 g. For solubility assay, cell pellets from small-scale expression cultures were resuspended in 500 μL of lysis buffer (Tris-HCl 50 mM, pH 7.5; NaCl 250 mM; EDTA 1 mM; lysosyme 1 mg mL^−1^; DNAse 0.1 mg ml^−1^) and incubated at 18 °C for 1 hour and the soluble and insoluble fractions separated by centrifugation (12000 g, 20 min, 4 °C). Soluble fractions were purified using His Microspin columns (GE Healthcare Life Science, USA) according to the protocol recommended by the supplier. The results were analyzed by 12% sodium dodecyl sulfate-polyacrylamide gel electrophoresis (SDS-PAGE). For the larger-scale production of the catalytic module of the ulvan lyase from *F*. *agariphila*, the cells were resuspended in a 20 mL of buffer A (20 mM Tris HCl pH 7.5, 500 mM NaCl and 5 mM Imidazole). An anti-proteases mixture (Complete EDTA-free, RocheTM) and 0.1 mg · mL^−1^ of DNase were added. The cells were disrupted in a French press. After centrifugation at 12500 g for 2 h at 4 °C, the supernatant was loaded onto a 10 mL Sepharose column (GE Healthcare) previously charged with NiSO4 100 mM and equilibrated with buffer A. The column was washed with buffer A (90 mL) and the protein was eluted with 60 mL of linear gradient between buffer A and buffer B (Tris HCl 20 mM pH 7.5; NaCl 500 mM; imidazole 500 mM) with a flow rate at 1 mL · min^−1^. The different fractions (1 mL each) were analyzed by SDS-PAGE. The fractions corresponding to a single band at the expected size (30.1 kDa) were pooled and were concentrated by ultrafiltration on an Amicon membrane (10 kDa-cutoff) (4.6 mL at 6.6 mg · mL^−1^). Ulvan lyase activity was assayed by measuring the increase in absorbance at 235 nm (A235) of the reaction products (unsaturated uronates) for 5 min in a 1-cm quartz cuvette containing 0.5 ml of reaction mixture in a thermostated spectrophotometer. The reaction mixture comprised 8 μL of ulvan lyase (0.66 μg · μL^−1^) and 492 μL of a solution of ulvan (0.55 μg · μL^−1^) with 100 mM MOPS buffer pH 7.5. Pure ulvan was purchased from Elycityl.

### Immunocytochemistry

The pea apices approximately 5 mm long were excised, fixed in 4% paraformaldehyde in PBS for 1 hour, washed with PBS twice and dehydrated through MeOH series. The MeOH was substituted by a 1:1 mixure of MeOH:(LR White resin) for 8 hours and finally in pure LR resin overnight. The polymerisation was performed at 60 °C overnight. The 1 μm-thick sections were generated on the Leica ultramicrotome EM-UC7 and adhered to the SuperFrost slides. The paraffin sections of mouse skeletal muscles (abcam) were deparaffinised with xylene and rehydrated through ethanol series. The sections were blocked with 5% milk powder (MP) in PBS for 30 minutes, probed with primary antibody at 1:10 dilution in 5% MP/PBS for 1 hour, washed three times with PBS and probed with secondary anti-rat antibody conjugated to Alexa Fluor 488 (Invitrogen) in 3% BSA/PBS at 1:300 dilution for 1 hour and washed three times with PBS. Calcofluor White (Sigma) at the final concentration 100 μg/ml and propidium iodide (Fluka) at the 1 μM final concentration were used as counterstain. The samples were finally mounted in CitiFluor (Agar Scientific) and scanned using laser scanning confocal microscope Leica SP5 using UV diode (405 nm), argon (488 nm) and helium-neon (543 nm) lasers. The pictures were processed with GIMP2 software.

The algae used in this study were collected from young cultures and fixed for 1 hour on ice in a solution of 1% paraformaldehyde/0.5% glutaraldehyde in natural seawater or 100 mM phosphate buffer with pH 7.8. The algae were then washed three times at 10 min intervals in ice cold seawater or 100 mM phosphate buffer. The algae were then dehydrated over 6 hours in an increasing series of cold EtOH (e.g. 10–30–50–70–90–100% EtOH) and infiltrated in cold 1:2, 1:1 and 2:1 London Resin (LR)-EtOH solutions over 24 hours. The algae were then placed in the base of beem capsules or microcentrifuge tubes and covered with pure LR with added accelerator. The LR was polymerized at 4 °C with UV light for 12 hours. 500 nm sections were then cut on a Leica Ultracut ultramicrotome and adhered to individual wells of a 10- or 12-welled poly-L-lysine coated, immunoslide (EMS). Immunolabeling with primary and secondary antibodies followed the protocol described above for pea sections. For some algae, a TRITC-conjugated secondary antibody was used instead of the Alexafluor 488-conjugated antibody in order to enhance contrast of the antibody labelling signal against chlorophyll autofluorescence. Images were acquired using an Olympus BX-60 LM equipped with fluorescence optics or an Olympus Fluoview 1200 confocal laser scanning microscope.

### TEM Microscopy

The algae were fixed on ice in 1% glutaraldehyde in 100 mM phosphate buffer (pH 7.8) for 1 h. After washing with 100 mM phosphate buffer three times, the algae were briefly post-fixed (1 h) on ice in 0.5% OsO_4_ in phosphate buffer. The algae were then washed in phosphate buffer, then deionized water and then dehydrated in an increasing concentration of acetone over 12 h. The algae were then embedded in Spurrs Resin and blocks were polymerized at 60 °C for 6 h. Ultrathin sections were obtained using a Reichert Ultracut ulramicrotome with a diamond knife. Sections were immunolabeled as previously described^53^ and observed with a Zeiss Libra 120 TEM.

### Surface plasmon resonance

*K*_D_ for matopentaose (Sigma) was determined by SPR (BIAcore T100; GE Healthcare) using streptavidin coated sensor chips (GE Healthcare) loaded with biotinylated INCh1 IgM (~2000 resonance units (RU)). Protein concentration prior to SPR was determined using an Abcam Mouse IgM ELISA Kit (ab133047) according to the protocol supplied by the manufacturer. INCh1 was buffer-exchanged to 10 mM HEPES, 150 mM NaCl, pH 7.5 using Amicon Ultra Centrifugal Filters (50 kDa molecular weight cut off, Millipore) and biotinylated with a 20-fold molar excess of EZ-link sulfo-NHS-LC-Biotin (Thermo Scientific) in the above buffer (30 min, RT). Excess reagent was removed (PD SpinTrap G-25 columns; GE Healthcare) while exchanging to running buffer (as above with 0.005% P20 surfactant added). Sensorgrams (RU vs. time) for maltopentaose (34–625 μM) were recorded at 25 °C in running buffer at 30 μl min^−1^, 60 s contact, 120 s dissociation and were baseline-corrected by subtracting data from a parallel flow cell with no INCh1 and normalized to the blank sensorgram. *K*_D_ was calculated by steady state affinity equation (BIAcore T100 evaluation 2.0.3; GE Healthcare) (eq. 1):

1 $$R=\frac{{R}_{{\rm{\max }}}[{\rm{Maltopentaose}}]}{[{\rm{Maltopentaose}}]+{K}_{{\rm{D}}}}$$

R is the resonance and R_max_ the maximum binding capacity in RU. Stoichiometry was calculated using (eq. 2):

2 $${\rm{stoichiometry}}=\frac{{{\rm{R}}}_{{\rm{\max }}}\cdot {{\rm{MW}}}_{{\rm{protein}}}}{{{\rm{MW}}}_{{\rm{\beta }}-{\rm{CD}}}\cdot {{\rm{R}}}_{{\rm{immobilized}}{\rm{protein}}}}$$

R_immobilized protein_ is in RU. Analyses were conducted in triplicates. See Fig. S7.

### Monosaccharide composition analysis

Monosaccharide composition analysis were performed as previously described in Øbro *et al*.^54^. Monosaccharide composition analysis was performed on the 2 h sequential extraction with CDTA, 4 M NaOH and the remaining pellet.

## Electronic supplementary material

Supplementary Information
