# Supplementary material for: Development of novel monoclonal antibodies against starch and ulvan - implications for antibody production against polysaccharides with limited immunogenicity
Source: Sci Rep. 2017 Aug 24;7:9326. doi: 10.1038/s41598-017-04307-2 (PMC5570955; doi:10.1038/s41598-017-04307-2)
Supplement: Supplementary file 1 — Supplementary Information [file 41598_2017_4307_MOESM1_ESM.pdf]

Supplementary Information

Development of novel monoclonal antibodies against starch and ulvan - implications for antibody production against polysaccharides with limited immunogenicity

Maja G. Rydahl*, Stjepan K. Kračun, Jonatan U. Fangel, Gurvan Michel, Alexia Guillouzo, Sabine Génicot, Jozef Mravec, Jesper Harholt, Casper Wilkens, Mohammed Saddik Motawia, Birte Svensson, Olivier Tranquet, Marie-Christine Ralet, Bodil Jørgensen, David S. Domozych and William G. T. Willats*

Figure S1. Characterisation of INCh1 and INCh2 using a microarray populated with diverse extracted polysaccharides from land plants.

Figure S2. Characterisation of INCh1 and INCh2 using a microarray populated with diverse CDTA extracted polysaccharides from algae.

Figure S2. Characterisation of INCh1 and INCh2 using a microarray populated with diverse NaOH extracted polysaccharides from algae.

Figure S4. Chemical structures of oligosaccharides used on microarrays in Figure 3

Figure S5. SPR sensogram

Figure S6. TEM of INCh1

Figure S7. Immunolabelling of glycogen in mouse muscle tissue using INCh1 and ECG1A9

Figure S8. Western blot and SDS-PAGE of ulvan lyase

Figure S9. Proposed schematic of immune overload

Figure S10. SDS-PAGE and Western blot analysis of INCh1 and INCh2 showing homogeneity of the respective antibodies

Table S1. Poly- and Oligosaccharide sample list

Table S2. Algae species in immunogen with growth conditions and growth media

Table S3. Assesment of secondary tagged anti-mouse IgG with IgM antibodies

Table S4. Enzymes used for epitope deletion

Figure S1

Extraction	Genus	Species	INCh1	INCh2	INCh3	INCh4	INCh5	INCh6	INCh7	INCh8	INCh9	INCh10	INCh11	INCh12	INCh13	INCh14	INCh15	INCh16	INCh17	INCh18	INCh19	INCh20	INCh21	INCh22	INCh23	INCh24	INCh25	INCh26	INCh27
CDTA	*Cornus*	*mas*	10	0	0	0	0	0	54	6	0	0	0	0	0	0	0	0	0	0	0	0	0	0	0	0	0	0	0
CDTA	*Arabidopsis*	*thaliana*	0	0	0	0	0	0	11	0	0	0	0	0	0	0	0	0	0	0	0	0	0	0	0	0	0	0	0
CDTA	*Viscum*	*album*	0	0	0	0	0	0	22	0	0	0	0	0	0	0	0	0	0	0	0	0	0	0	0	0	0	0	0
CDTA	*Brachypodium*	*distachyon*	0	0	0	0	0	0	0	0	0	0	0	0	0	0	0	0	0	0	0	0	0	0	0	0	0	0	0
CDTA	*Oryza*	*sativa*	0	0	0	0	0	0	0	0	0	0	0	0	0	0	0	0	0	0	0	0	0	0	0	0	0	0	0
CDTA	*Equisetum*	*arvense*	0	0	0	0	0	0	0	0	0	0	0	0	0	0	0	0	0	0	0	0	0	0	0	0	0	0	0
CDTA	*Selaginella*	*kraussiana*	0	0	0	0	0	0	0	0	0	0	0	0	0	0	0	0	0	0	0	0	0	0	0	0	0	0	0
CDTA	*Huperzia*	*phlegmaria*	0	0	0	0	0	0	0	43	0	0	0	0	0	0	0	0	0	0	0	0	0	0	0	0	0	0	33
CDTA	*Physcomitrella*	*patens*	0	0	0	0	0	0	3	0	0	0	0	0	0	0	0	0	0	0	0	0	0	0	0	0	0	0	0
CDTA	*Leiomylia*	*anomala*	0	0	0	0	0	0	0	0	0	0	0	0	0	0	0	0	0	0	0	0	0	0	0	0	0	0	0
CDTA	*Marchantia*	*polymorpha*	0	0	0	0	0	0	16	0	0	0	0	0	0	0	0	0	0	0	0	0	0	0	0	0	0	0	0
NaOH	*Cornus*	*mas*	58	0	0	0	0	0	50	6	0	17	0	0	0	0	0	0	0	0	0	0	0	0	0	0	0	0	0
NaOH	*Arabidopsis*	*thaliana*	21	0	0	0	0	0	7	0	0	0	0	0	0	0	0	0	0	0	0	0	0	0	0	0	0	0	0
NaOH	*Viscum*	*album*	49	0	0	0	0	0	6	0	0	14	20	0	0	0	0	0	0	0	0	0	0	0	0	0	0	0	0
NaOH	*Brachypodium*	*distachyon*	30	0	0	0	0	0	0	0	0	4	0	0	0	0	0	0	0	0	0	0	0	0	0	0	0	0	0
NaOH	*Oryza*	*sativa*	0	0	0	0	0	0	0	0	0	0	0	0	0	0	0	0	0	0	0	0	0	0	0	0	0	0	0
NaOH	*Equisetum*	*arvense*	23	0	0	0	0	0	0	0	0	0	0	0	0	0	0	0	0	0	0	0	0	0	0	0	0	0	0
NaOH	*Selaginella*	*kraussiana*	7	0	0	0	0	0	0	0	0	0	0	0	0	0	0	0	0	0	0	0	0	0	0	0	0	0	0
NaOH	*Huperzia*	*phlegmaria*	9	0	0	0	0	0	0	100	0	0	0	0	0	0	0	0	0	0	0	0	0	0	0	0	0	0	97
NaOH	*Physcomitrella*	*patens*	16	0	0	0	0	0	24	0	0	0	0	0	0	0	0	0	0	0	0	0	0	0	0	0	0	0	0
NaOH	*Leiomylia*	*anomala*	15	0	0	0	0	0	5	0	0	0	0	0	0	0	0	0	0	0	0	0	0	0	0	0	0	0	0
NaOH	*Marchantia*	*polymorpha*	31	0	0	0	0	0	5	0	0	6	0	0	0	0	0	0	0	0	0	0	0	0	0	0	0	0	0

Extraction	Genus	Species	HG with a low DE (mAb JIM5)	Backbone RGI (mAb INRA-RU1)	(1-4)-β-D-galactan (mAb LM5)	(1-5)-α-L-arabinan (mAb LM6)	Linearised (1-5)-α-L-arabinan (mAb LM13)	(1-4)-β-D-mannan (mAb LM21)	(1-4)-β-D-xylan (mAb LM10)	Xyloglucan (XXXG motif) (mAb LM15)	Feruloylate on any polymer (mAb LM12)	AGP (mAb JIM13)	Extensin (mAb JIM12)
CDTA	*Cornus*	*mas*	100	48	51	58	23	0	0	0	0	26	0
CDTA	*Arabidopsis*	*thaliana*	82	57	19	35	20	0	0	6	0	25	6
CDTA	*Viscum*	*album*	25	15	16	28	25	0	0	0	0	67	45
CDTA	*Brachypodium*	*distachyon*	27	0	6	13	0	0	0	0	33	18	0
CDTA	*Oryza*	*sativa*	24	0	0	5	0	0	0	0	22	17	0
CDTA	*Equisetum*	*arvense*	62	8	30	39	0	28	0	0	0	0	0
CDTA	*Selaginella*	*kraussiana*	0	0	0	7	0	69	0	0	0	14	0
CDTA	*Huperzia*	*phlegmaria*	0	0	0	0	0	60	0	13	0	0	0
CDTA	*Physcomitrella*	*patens*	24	0	10	51	0	0	0	0	0	34	6
CDTA	*Leiomylia*	*anomala*	0	0	0	0	0	9	0	0	5	32	0
CDTA	*Marchantia*	*polymorpha*	21	0	0	0	0	9	0	0	0	26	0
NaOH	*Cornus*	*mas*	0	12	59	35	9	39	68	55	6	42	0
NaOH	*Arabidopsis*	*thaliana*	0	12	13	8	0	9	0	18	0	23	0
NaOH	*Viscum*	*album*	0	16	0	13	25	37	52	59	0	48	26
NaOH	*Brachypodium*	*distachyon*	0	15	24	34	9	0	30	22	0	21	0
NaOH	*Oryza*	*sativa*	0	10	8	28	5	0	6	18	0	21	0
NaOH	*Equisetum*	*arvense*	0	14	29	28	5	10	0	5	0	0	0
NaOH	*Selaginella*	*kraussiana*	17	0	13	23	0	19	0	18	0	13	0
NaOH	*Huperzia*	*phlegmaria*	0	0	14	0	0	19	6	29	0	0	0
NaOH	*Physcomitrella*	*patens*	0	33	9	23	0	18	0	0	0	0	0
NaOH	*Leiomylia*	*anomala*	0	0	40	11	0	45	0	0	0	49	0
NaOH	*Marchantia*	*polymorpha*	0	22	0	9	0	46	0	0	0	58	0

Flowering plants **Ferns** **Clubmoss** **Moss** **Liverworts**

0 10 20 30 40 50 60 70 80 90 100

Figure S1. Characterisation of INCh1 and INCh2 using a microarray populated with diverse extracted polysaccharides from land plants. Heatmap showing binding analysis of INCh1, INCh2 and 11 control mAbs using extracted carbohydrate microarrays (Comprehensive microarray polymer profiling (CoMPP)). The heatmap shows the relative abundance of cell wall components as extracted using CDTA and NaOH. Arrays were populated with polysaccharides from diverse land plant sources. Mean spot signals obtained are presented in a heatmap in which colour intensity is correlated to signal intensity. The highest signal in each dataset was set to 100, and all other values were normalized accordingly as indicated by the colour scale bar. A low-end cut-off value of 5 was used. The same amount of cell wall material (alcohol insoluble residue) was used for each sample. Phylums of the individual species are indicated according to the colour codes. Homogalacturonan (HG), arabinogalactan protein (AGP), rhamnogalacturonan (RG).

Figure S2. Characterisation of INCh1 and INCh2 using a microarray populated with diverse CDTA extracted poly-saccharides from algae. Heatmap showing analysis of INCh1, INCh2 and 11 control mAbs binding using extracted carbohydrate microarrays (Comprehensive microarray polymer profiling (CoMPP)). The heatmap shows the relative abundance of cell wall components as extracted using CDTA. Arrays where populated with polysaccharides from diverse algal sources. Mean spot signals obtained are presented in a heatmap in which color intensity is corre-lated to signal. The highest signal in each dataset was set to 100, and all other values were normalized accordingly as indicated by the color scale bar. A low-end cut-off value of 5 was used. The same amount of cell wall material (alcohol insoluble residue) was used for each sample. Classes of the individual species are indicated according to the colour codes. Homogalacturonan (HG), arabinogalactan protein (AGP), rhamnogalacturonan (RG).

Figure S2

CDTA

Genus	Species	INCh1	INCh2	INCh3	INCh4	INCh5	INCh6	INCh7	INCh8	INCh9	INCh10	INCh11	INCh12	INCh13	INCh14	INCh15	INCh16	INCh17	INCh18	INCh19	INCh20	INCh21	INCh22	INCh23	INCh24	INCh25	INCh26	INCh27	HG with a low DE (mAb JIM5)	Backbone RGI (mAb INRA-RU1)	(1-4)-β-D-galactan (mAb LM5)	(1-5)-α-L-arabinan (mAb LM6)	Linearised (1-5)-α-L-arabinan (mAb LM13)	(1-4)-β-D-mannan (mAb LM21)	(1-4)-β-D-xylan (mAb LM10)	Xyloglucan (XXXG motif) (mAb LM15)	Feruloylate on any polymer (mAb LM12)	AGP (mAb JIM13)	Extensin (mAb JIM12)
Closterium	*acerosum*	6	0	0	0	0	0	0	24	0	0	0	0	0	0	0	0	0	0	0	0	0	0	0	0	0	0	0	0	0	0	0	0	0	0	0	0	54	0
Closterium	*moniliforma*	0	0	0	0	0	0	0	0	0	0	0	0	0	0	0	0	0	0	0	0	0	0	0	0	0	0	0	0	0	0	0	0	0	0	0	0	45	0
Closterium	*didymo*	0	0	0	0	0	0	0	0	0	0	0	0	0	0	0	0	0	0	0	0	0	0	0	0	0	0	0	0	0	0	0	0	0	0	0	0	0	0
Cosmarium	*botrytis*	0	0	0	0	0	0	0	0	0	0	0	0	0	0	0	0	0	0	0	0	0	0	0	0	0	0	0	0	0	0	0	0	0	0	0	0	0	0
Micrasterias	*furcata*	15	0	0	0	0	0	16	9	0	11	0	0	0	0	0	0	0	0	0	0	0	0	0	0	0	0	0	0	5	0	0	0	0	0	0	0	46	0
Teilingia	*granulata*	22	0	0	0	0	0	16	12	0	10	0	0	0	0	0	0	0	0	0	0	0	0	0	0	0	0	0	0	0	0	0	0	0	0	0	0	0	0
Penium	*margaritaceum*	8	0	0	0	0	0	0	32	0	7	0	0	0	0	0	0	0	0	0	0	0	0	0	0	0	0	0	10	35	0	0	0	0	0	0	0	6	0
Groenbladia	*sp.*	0	0	0	0	0	0	11	66	0	0	0	0	0	0	0	0	0	0	0	0	0	0	0	0	0	0	0	0	0	0	29	0	0	0	0	0	0	0
Pleurotaenium	*trabecula*	30	0	0	0	0	0	22	0	0	22	0	0	0	0	0	0	0	0	0	0	0	0	0	0	0	0	0	0	0	0	0	0	0	0	0	0	22	0
Cylindrocystis	*sp.*	0	0	0	0	0	0	18	22	0	0	0	0	0	0	0	0	0	0	0	0	0	0	0	0	0	0	0	0	0	0	23	43	0	0	0	0	33	0
Mesotaenium	*caldariorum*	0	0	0	0	0	0	0	8	17	0	0	0	0	0	0	0	0	0	0	0	0	0	0	0	0	0	0	0	0	0	0	0	0	0	0	0	0	0
Mougeotia	*transeau*	7	0	0	0	0	0	29	28	0	0	0	0	0	0	0	0	0	0	0	0	0	0	0	0	0	0	13	0	0	0	0	0	0	0	0	0	0	0
Spirogyra	*maxima*	44	0	0	0	0	0	16	0	0	32	0	0	0	0	0	0	0	0	0	0	0	0	0	0	0	0	0	0	74	0	0	0	0	0	0	0	30	0
Spirogyra	*communis*	0	0	0	0	0	0	8	0	19	0	0	0	0	0	0	0	0	0	0	0	0	0	0	0	0	0	0	0	18	0	0	0	0	0	0	0	38	0
Spirogyra	*praetensis*	36	0	0	0	0	0	8	0	0	29	0	0	0	0	0	0	0	0	0	0	0	0	0	0	0	0	0	0	0	0	0	0	0	0	0	0	21	0
Netrium	*interruptum*	0	0	0	0	0	0	0	0	0	0	0	0	0	0	0	0	0	0	0	0	0	0	0	0	0	0	0	29	0	0	0	0	0	0	0	0	0	0
Roya	*sp.*	7	0	0	0	0	0	0	6	0	0	0	0	0	0	0	0	0	0	0	0	0	0	0	0	0	0	0	21	69	0	0	0	0	0	0	0	11	0
Coleochaete	*nitellarum*	0	0	0	0	0	0	0	10	0	0	0	0	0	0	0	0	0	0	0	0	0	0	0	0	0	0	0	9	5	0	0	0	5	0	0	0	0	0
Chaetosphaeridium	*sp.*	13	0	0	0	0	0	0	3	0	11	0	0	0	0	0	0	0	0	0	0	0	0	0	0	0	0	0	0	9	0	0	0	0	0	0	0	21	0
Coleochaete	*orbicularis*	0	0	0	0	5	0	21	22	0	0	0	0	0	0	0	0	0	0	0	0	0	0	0	0	0	0	0	5	41	0	29	0	0	0	0	0	44	0
Chara	*corallina*	9	0	0	0	0	0	19	31	0	8	0	0	0	0	0	0	0	0	0	0	0	0	0	0	0	0	7	0	21	0	0	0	0	0	0	0	18	0
Klebsormidium	*flaccidum*	0	0	0	0	0	0	0	0	0	0	0	0	0	0	0	0	0	0	0	0	0	0	0	0	0	0	0	0	0	0	0	0	0	0	0	0	0	0
Klebsormidium	*dissectum*	0	0	0	0	0	0	0	0	0	0	0	0	0	0	0	0	0	0	0	0	0	0	0	0	0	0	0	0	0	0	6	0	0	0	0	0	0	0
Mesostigma	*sp.*	0	0	0	0	0	0	0	0	0	0	0	0	0	0	0	0	0	0	0	0	0	0	0	0	0	0	0	0	0	0	0	0	0	0	0	0	0	0
Chlorokybus	*atmophyticus*	0	0	0	0	0	0	0	0	0	0	0	0	0	0	0	0	0	0	0	0	0	0	0	0	0	0	0	0	0	0	0	0	0	0	0	0	32	0
Dolichomastix	*tenuilepis*	12	0	0	0	0	0	0	0	0	7	0	0	0	0	0	0	0	0	0	0	0	0	0	0	0	0	0	0	0	0	0	0	0	0	0	0	0	0
Pseudoscourfieldia	*marina*	11	0	0	0	0	0	0	0	0	7	0	0	0	0	0	0	0	0	0	0	0	0	0	0	0	0	0	0	0	0	0	0	0	0	0	0	0	0
Tetraselmis	*striata*	0	0	0	0	0	0	0	0	0	0	0	0	0	0	0	0	0	0	0	0	0	0	0	0	0	0	0	0	9	0	0	0	0	0	0	0	0	0
Chlorella	*sp.*	0	0	0	35	33	58	0	0	0	0	25	0	0	0	0	0	0	0	0	0	0	0	0	0	0	0	0	0	0	0	5	0	0	0	0	0	0	0
Pedinomonas	*minor*	13	0	0	0	0	0	0	0	0	7	0	0	0	0	0	0	0	0	0	0	0	0	0	0	0	0	0	0	9	0	0	0	0	0	0	0	0	0
Oedogonium	*foveolatum*	0	0	0	0	0	0	0	0	0	0	0	0	0	0	0	0	0	0	0	0	0	0	0	0	0	0	0	0	0	0	0	0	6	0	0	0	8	0
Chlamydomonas	*reinhardii*	0	0	0	0	0	0	0	0	0	0	0	0	0	0	0	0	0	0	0	0	0	0	0	0	0	0	0	0	0	0	0	0	0	0	0	0	0	0
Haematococcus	*pluvialis*	0	0	0	0	0	0	0	0	0	0	0	0	0	0	0	0	0	0	0	0	0	0	0	0	0	0	0	0	0	0	0	0	8	0	0	0	0	0
Pandorina	*sp.*	13	0	0	0	0	0	12	0	0	9	0	0	0	0	0	0	0	0	0	0	0	0	0	0	0	0	0	0	0	0	0	0	0	0	0	0	0	0
Dunaliella	*tertiolecta*	8	0	0	0	0	0	0	0	0	0	0	0	0	0	0	0	0	0	0	0	0	0	0	0	0	0	0	0	7	0	0	0	0	0	0	0	0	0
Scenedesmus	*quadricauda*	0	0	0	0	0	0	0	0	0	0	0	0	0	0	0	0	0	0	0	0	0	0	0	0	0	0	0	0	0	0	0	0	0	0	0	0	0	0
Microspora	*sp.*	9	0	0	0	0	0	21	41	0	0	0	0	0	0	0	0	0	0	0	0	0	0	0	0	0	0	20	0	0	0	0	0	0	0	0	0	18	0
Hydrodictyon	*sp.*	0	0	0	0	0	0	0	0	0	0	0	0	0	0	0	0	0	0	0	0	0	0	0	0	0	0	0	0	0	0	0	0	0	0	0	0	0	0
Caulerpa	*microphysa*	0	0	0	0	0	0	65	0	0	0	0	0	0	0	0	0	0	0	0	0	0	0	0	0	0	0	0	0	0	0	0	0	0	0	0	0	0	0
Caulerpa	*taxafolia*	0	0	0	0	0	0	98	0	0	0	0	0	0	0	0	0	0	0	0	0	0	0	0	0	0	0	0	0	0	0	0	0	0	0	0	0	0	0
Codium	*sp.*	0	0	0	0	0	0	0	0	0	0	0	0	0	5	0	0	7	0	0	0	0	0	0	0	0	0	0	0	9	0	0	0	5	0	0	0	0	0
Blastophysa	*rhizopus*	11	0	0	0	0	0	14	0	0	0	0	0	0	0	0	0	0	0	0	0	0	0	0	0	0	0	0	0	0	0	0	0	0	0	0	0	0	0
Cladophora	*glomerata*	15	0	0	0	0	7	7	0	0	7	19	0	0	11	6	5	14	0	0	5	5	0	0	0	0	0	0	0	7	0	0	0	0	0	0	0	0	0
Interfilum	*2167*	0	0	0	0	0	0	0	0	83	0	0	0	0	0	0	0	0	0	0	0	0	0	0	0	0	0	0	0	0	0	26	0	0	0	0	0	0	0
Ulothrix	*fimbrata*	16	0	0	0	0	0	0	0	0	6	0	0	0	0	0	0	0	0	0	0	0	0	0	0	0	0	0	0	0	0	0	0	0	0	0	0	0	0
Pseudendoclonium	*sp.*	0	0	0	0	0	5	0	0	0	0	14	8	0	18	10	9	22	0	5	6	9	9	0	0	0	9	0	5	19	5	0	0	0	20	0	0	5	0
Epicladia	*sp.*	0	0	0	0	0	0	0	22	0	0	0	0	0	0	0	0	0	0	0	0	0	0	0	0	0	0	7	0	0	0	0	0	0	10	0	0	31	0
Ulvella	*sp.*	0	0	0	0	0	0	12	20	0	0	0	0	0	0	0	0	0	0	0	0	0	0	0	0	0	0	8	0	0	0	0	0	0	0	0	0	13	0
Ulva	*lactuta*	0	100	0	0	0	0	21	26	0	0	0	0	0	0	0	0	0	0	0	0	0	0	0	0	0	0	9	0	0	0	0	0	0	0	0	0	0	0
Enteromorpha	*intestinalis*	0	62	0	0	0	0	34	38	0	0	0	0	0	0	0	0	0	0	0	0	0	0	0	0	0	0	31	0	0	0	0	0	0	0	0	0	12	0

Chlorophyta | Charophyta | Core chlorophytes | Prasinophytes | Ulvophyceae | Chlorophyceae | Trebouxiophyceae

0 | 10 | 20 | 30 | 40 | 50 | 70 | 90 | 100

Figure S3. Characterisation of INCh1 and INCh2 using a microarray populated with diverse NaOH extracted polysaccharides from algae. Heatmap showing analysis of INCh1, INCh2 and 11 control mAbs binding using extracted carbohydrate microarrays (Comprehensive microarray polymer profiling (CoMPP)). The heatmap shows the relative abundance of cell wall components as extracted using NaOH. Arrays where populated with polysaccharides from diverse algal sources. Mean spot signals obtained are presented in a heatmap in which colour intensity is correlated to signal. The highest signal in each dataset was set to 100, and all other values were normalized accordingly as indicated by the colour scale bar. A low-end cut-off value of 5 was used. The same amount of cell wall material (alcohol insoluble residue) was used for each sample. Classes of the individual species are indicated according to the colour codes. Homogalacturonan (HG), arabinogalactan protein (AGP), rhamnogalacturonan (RG).

Figure S3

NaOH

Genus	Species	INCh1	INCh2	INCh3	INCh4	INCh5	INCh6	INCh7	INCh8	INCh9	INCh10	INCh11	INCh12	INCh13	INCh14	INCh15	INCh16	INCh17	INCh18	INCh19	INCh20	INCh21	INCh22	INCh23	INCh24	INCh25	INCh26	INCh27	HG with a low DE (mAb JIM5)	Backbone RGI (mAb INRA-RU1)	(1-4)-β-D-galactan (mAb LM5)	(1-5)-α-L-arabinan (mAb LM6)	Linearised (1-5)-α-L-arabinan (mAb LM13)	(1-4)-β-D-mannan (mAb LM21)	(1-4)-β-D-xylan (mAb LM10)	Xyloglucan (XXXG motif) (mAb LM15)	Feruloylate on any polymer (mAb LM12)	AGP (mAb JIM13)	Extensin (mAb JIM12)
Closterium	*acerosum*	40	0	0	0	0	0	0	20	0	21	0	0	0	0	0	0	0	0	0	0	0	0	0	0	0	0	0	0	0	0	0	0	0	0	0	0	50	0
Closterium	*moniliforma*	19	0	0	0	0	0	6	44	0	9	0	5	0	0	0	0	0	0	0	0	0	0	0	0	0	0	39	0	0	0	0	0	0	0	0	0	35	0
Closterium	*didymo*	59	0	0	0	0	0	0	53	0	46	0	11	0	0	0	0	0	0	0	0	0	0	0	0	0	0	43	0	0	0	0	0	0	0	0	0	37	0
Cosmarium	*botrytis*	9	0	0	0	0	0	6	25	0	0	0	0	0	0	0	0	0	0	0	0	0	0	0	0	0	0	0	0	0	0	0	0	0	0	0	0	0	0
Micrasterias	*furcata*	16	0	0	0	0	0	5	0	0	5	0	0	0	0	0	0	0	0	0	0	0	0	0	0	0	0	0	0	0	0	0	0	0	0	0	0	24	0
Teilingia	*granulata*	38	0	0	0	0	0	34	27	0	30	0	7	0	0	0	0	0	0	0	0	0	0	0	0	0	0	0	0	9	0	0	0	0	0	0	0	0	0
Penium	*margaritaceum*	74	0	0	0	0	0	0	43	0	57	0	0	0	0	0	0	0	0	0	0	0	0	0	0	0	0	22	0	29	0	0	0	0	0	0	0	0	0
Groenbladia	*sp.*	20	0	0	0	0	0	18	23	0	11	0	0	0	0	0	0	0	0	0	0	0	0	0	0	0	0	0	0	0	0	10	0	0	0	0	0	0	0
Pleurotaenium	*trabecula*	53	0	0	0	0	0	13	0	0	40	0	0	0	0	0	0	0	0	0	0	0	0	0	0	0	0	0	0	4	0	0	0	0	0	0	0	20	0
Cylindrocystis	*sp.*	32	0	0	0	0	0	16	0	0	13	0	0	0	0	0	0	0	0	0	0	0	0	0	0	0	0	0	0	33	0	21	22	0	0	0	0	20	0
Mesotaenium	*caldariorum*	29	0	0	0	0	0	0	9	32	0	0	0	0	0	0	0	0	0	0	0	0	0	0	0	0	0	0	0	8	0	0	0	0	0	0	0	0	0
Mougeotia	*transeau*	12	0	0	0	0	0	9	23	0	6	0	0	0	0	0	0	0	0	0	0	0	0	0	0	0	0	0	0	12	0	0	0	0	0	0	0	0	0
Spirogyra	*maxima*	48	0	0	0	0	0	14	0	0	36	0	7	0	0	0	0	0	0	0	0	0	0	0	0	0	0	0	0	13	0	0	0	0	0	0	0	9	0
Spirogyra	*communis*	46	0	0	0	0	0	0	0	12	27	0	0	0	0	0	0	0	0	0	0	0	0	0	0	0	0	0	0	28	0	0	0	0	0	0	0	18	0
Spirogyra	*praetensis*	48	0	0	0	0	0	5	0	0	36	0	0	0	0	0	0	0	0	0	0	0	0	0	0	0	0	0	0	0	0	0	0	0	0	0	0	0	0
Netrium	*interruptum*	66	0	0	0	0	0	0	0	0	49	0	0	0	0	0	0	0	0	0	0	0	0	0	0	0	0	0	0	0	0	21	0	0	0	10	0	0	0
Roya	*sp.*	15	0	0	0	0	0	0	0	0	0	0	0	0	0	0	0	0	0	0	0	0	0	0	0	0	0	0	0	14	0	0	0	0	0	0	0	13	0
Coleochaete	*nitellarum*	22	0	0	0	0	0	0	0	0	6	0	0	0	0	0	0	0	0	0	0	0	0	0	0	0	0	0	0	0	0	0	0	10	0	0	0	0	0
Chaetosphaeridium	*sp.*	23	0	0	0	0	0	0	0	0	15	0	8	0	0	0	0	0	0	0	0	0	0	0	0	0	0	0	7	32	0	0	0	8	0	0	0	21	0
Coleochaete	*orbicularis*	9	0	0	0	0	0	25	31	0	0	0	5	0	0	0	0	0	0	0	0	0	0	0	0	0	0	0	0	20	0	46	0	0	0	0	0	39	0
Chara	*corallina*	41	0	0	0	0	0	15	23	0	16	0	0	0	0	0	0	0	0	0	0	0	0	0	0	0	0	11	0	31	0	0	0	0	0	0	0	20	0
Klebsormidium	*flaccidum*	24	0	0	0	0	0	0	0	0	0	0	0	0	0	0	0	0	0	0	0	0	0	0	0	0	0	0	0	0	0	0	0	0	0	0	0	0	0
Klebsormidium	*dissectum*	6	0	0	0	0	0	0	0	0	0	0	0	0	0	0	0	0	0	0	0	0	0	0	0	0	0	0	0	0	0	0	0	0	0	0	0	0	0
Mesostigma	*sp.*	9	0	0	0	0	0	0	0	0	0	0	6	0	0	0	0	0	0	0	0	0	0	0	0	0	0	0	0	0	0	0	0	3	0	0	0	0	0
Chlorokybus	*atmophyticus*	24	0	0	0	0	0	0	0	0	0	0	5	0	0	0	0	0	0	0	0	0	0	0	0	0	0	0	0	0	0	0	0	0	0	0	0	16	0
Dolichomastix	*tenuilepis*	11	0	0	0	0	0	0	0	0	0	0	0	0	0	0	0	0	0	0	0	0	0	0	0	0	0	0	0	0	0	0	0	0	0	0	0	0	0
Pseudoscourfieldia	*marina*	12	0	0	0	0	0	0	0	0	0	0	0	0	0	0	0	0	0	0	0	0	0	0	0	0	0	0	0	0	0	0	0	0	0	0	0	0	0
Tetraselmis	*striata*	0	0	0	0	0	0	0	0	0	0	0	0	0	0	0	0	0	0	0	0	0	0	0	0	0	0	0	0	0	0	0	0	0	0	0	0	0	0
Chlorella	*sp.*	5	0	0	0	0	0	0	0	0	0	0	0	0	0	0	0	0	0	0	0	0	0	0	0	0	0	0	0	0	0	6	0	10	0	0	0	0	0
Pedinomonas	*minor*	15	0	0	0	0	0	0	0	0	10	0	0	0	0	0	0	0	0	0	0	0	0	0	0	0	0	0	0	12	0	0	0	0	0	0	0	0	0
Oedogonium	*foveolatum*	43	0	0	0	0	0	0	7	0	19	0	5	0	0	0	0	0	0	0	0	0	0	0	0	0	0	0	0	0	0	0	0	17	0	0	0	8	0
Chlamydomonas	*reinhardii*	13	0	0	0	0	0	0	0	0	0	0	0	0	0	0	0	0	0	0	0	0	0	0	0	0	0	0	0	0	0	0	0	0	0	0	0	0	0
Haematococcus	*pluvialis*	18	0	0	0	0	0	0	0	0	13	0	0	0	0	0	0	0	0	0	0	0	0	0	0	0	0	0	0	0	0	0	0	39	0	0	0	0	0
Pandorina	*sp.*	9	0	0	0	0	0	0	0	0	5	0	0	0	0	0	0	0	0	0	0	0	0	0	0	0	0	0	0	0	0	0	0	0	0	0	0	0	0
Dunaliella	*tertiolecta*	13	0	0	0	0	0	0	0	0	0	0	0	0	0	0	0	0	0	0	0	0	0	0	0	0	0	0	0	0	0	0	0	0	0	0	0	0	0
Scenedesmus	*quadricauda*	32	0	0	0	0	0	0	0	0	27	0	0	0	0	0	0	0	0	0	0	0	0	0	0	0	0	0	0	0	0	0	0	34	0	0	0	0	0
Microspora	*sp.*	30	0	0	0	0	0	8	16	0	20	0	11	0	0	0	0	0	0	0	0	0	0	0	0	0	0	0	0	0	0	0	0	7	0	0	0	5	0
Hydrodictyon	*sp.*	39	0	0	0	0	0	0	0	0	28	0	0	0	0	0	0	0	0	0	0	0	0	0	0	0	0	0	0	0	0	0	0	21	0	0	0	12	0
Caulerpa	*microphysa*	26	5	0	0	0	0	60	0	18	10	0	0	0	0	0	0	0	0	0	0	0	0	0	0	0	0	0	0	6	0	0	0	0	0	0	0	0	0
Caulerpa	*taxafolia*	45	0	0	0	0	0	95	0	0	27	0	0	0	0	0	0	0	0	0	0	0	0	0	0	0	0	0	0	0	0	0	0	0	0	0	0	0	0
Codium	*sp.*	37	0	0	0	0	0	0	0	0	29	0	0	0	0	0	0	5	0	0	0	0	0	0	0	0	0	0	0	6	0	0	0	37	0	0	0	0	0
Blastophysa	*rhizopus*	35	0	0	0	0	0	5	0	0	20	0	0	0	0	0	0	0	0	0	0	0	0	0	0	0	0	0	0	0	0	0	0	0	0	0	0	0	0
Cladophora	*glomerata*	42	8	0	0	0	0	0	0	0	23	7	0	0	0	0	0	6	0	0	0	0	0	0	0	0	0	0	0	7	0	0	0	0	0	0	0	0	0
Interfilum	*2167*	5	0	0	0	0	0	0	0	67	0	0	0	0	0	0	0	0	0	0	0	0	0	0	0	0	0	0	0	0	0	8	0	3	0	0	0	0	0
Ulothrix	*fimbrata*	27	0	0	0	0	0	0	0	0	16	0	0	0	0	0	0	0	0	0	0	0	0	0	0	0	0	0	0	0	0	0	0	17	0	0	0	0	0
Pseudendoclonium	*sp.*	16	5	0	0	0	0	0	0	0	6	5	0	0	9	5	5	10	0	0	0	0	0	0	0	0	0	0	0	13	0	0	0	0	20	0	0	6	0
Epicladia	*sp.*	16	5	0	0	0	0	0	9	0	11	0	0	0	0	0	0	0	0	0	0	0	0	0	0	0	0	0	0	0	0	0	0	0	8	0	0	31	0
Ulvella	*sp.*	31	0	0	0	0	0	0	0	0	17	0	0	0	0	0	0	0	0	0	0	0	0	0	0	0	0	0	0	0	0	0	0	0	0	0	0	0	0
Ulva	*lactuta*	11	63	0	0	0	0	0	0	0	0	0	0	0	0	0	0	0	0	0	0	0	0	0	0	0	0	0	0	0	0	0	0	0	0	0	0	0	0
Enteromorpha	*intestinalis*	11	49	0	0	0	0	6	0	0	0	0	0	0	0	0	0	0	0	0	0	0	0	0	0	0	0	0	0	0	0	0	0	0	0	0	0	0	0

Chlorophyta **Charophyta** **Core chlorophytes** **Prasinophytes** **Ulvophyceae** **Chlorophyceae** **Trebouxiophyceae**

0 10 20 30 40 50 70 90 100

Figure S4

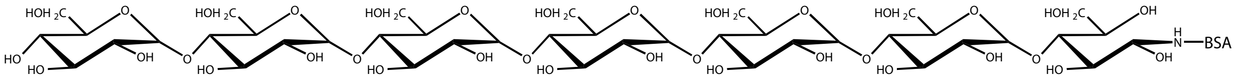

1

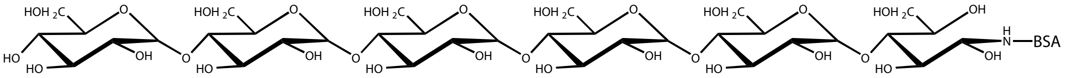

2

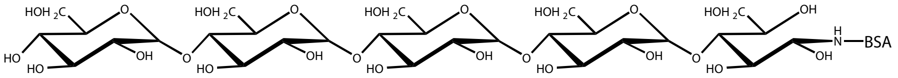

3

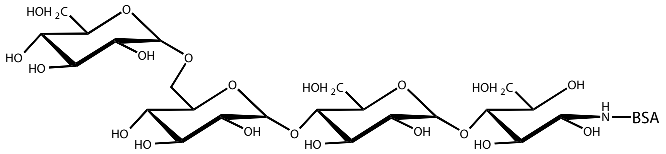

4

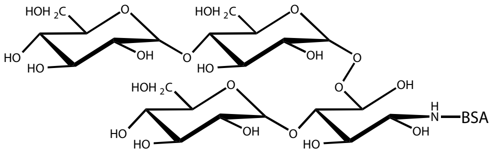

5

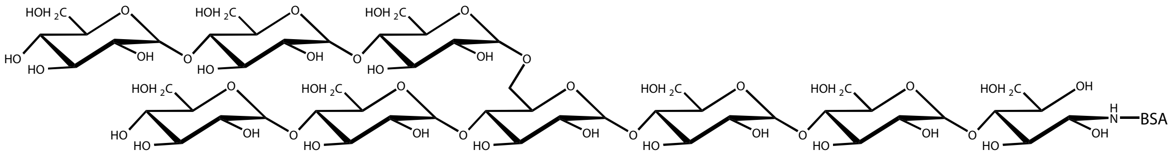

6

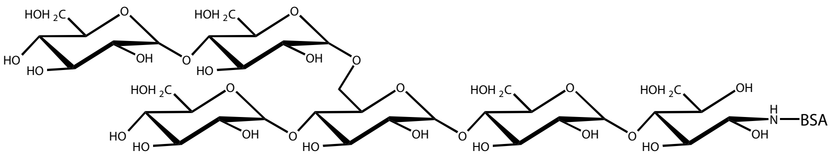

7

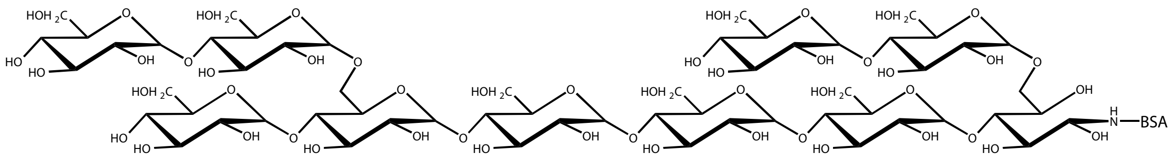

8

Figure S4. Chemical structures of oligosaccharides used on microarrays in Figure 3
Structure: 1) maltoheptaose, 2) maltohexaose, 3) maltopentaose, 4) α-(1-6)-D-glucosyl-maltotriose, 5) 6^{1}-α-maltosyl-maltose, 6) 6^{4}-α-maltotriosyl-maltohexaose, 7) 6^{3}-α-maltosyl-maltotetraose, 8) 6^{1},6^{5}-di-α-maltosyl-maltohexaose.

Figure S5

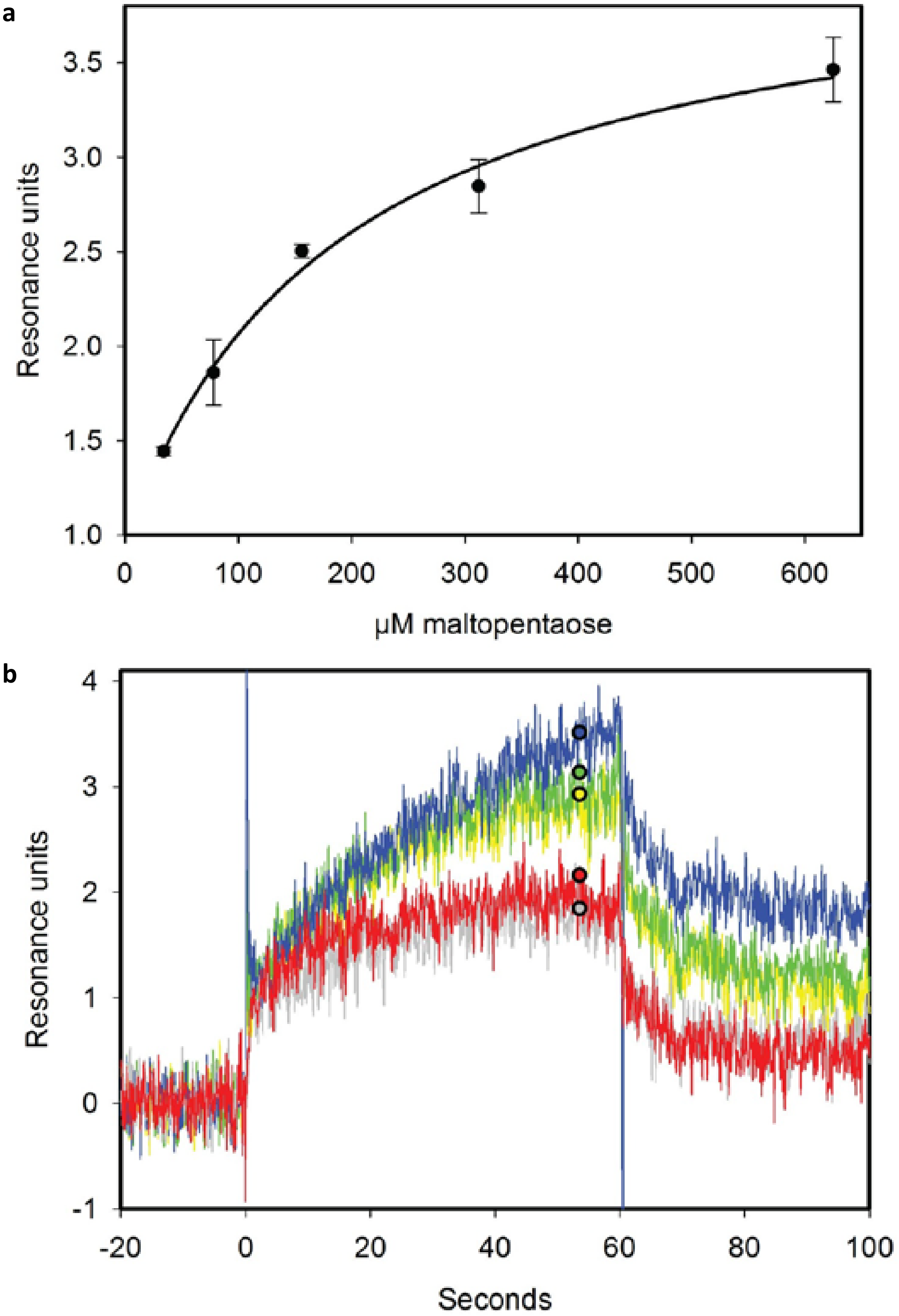

Figure S5. SPR sensorgram

a) Plots for determination of K_D for maltopentaose binding to IgM calculated according to eq. 1 (see Materials and Methods). The individual data points were derived from the sensorgrams shown in Figure S6b. **b)** Representative sensorgrams for maltopentaose SPR analysis of IgM. The points used for the steady-state fit (Figure S7a) are marked with a ○. 34 µM is grey, 78 µM is red, 156 µM is yellow, 312 µM is green and 625 µM is blue.

Figure S6

Chlorophytes

Caulerpa microphysa
(Ulvophyceae)

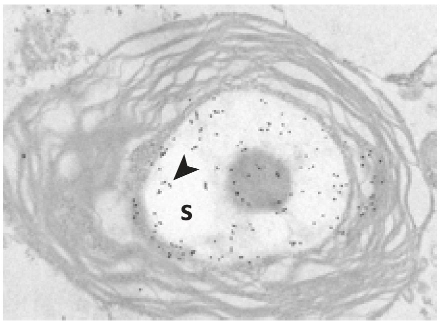

Charophytes

Penium margaritaceum
(Zygnematophyceae)

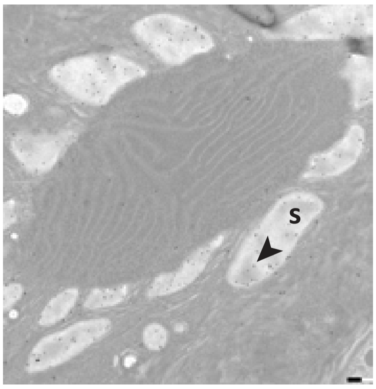

Penium margaritaceum
(Zygnematophyceae)

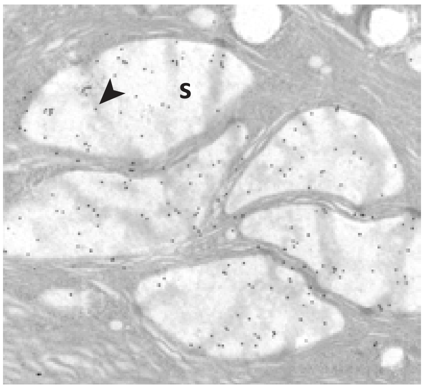

Coleochaete orbicularis
(Coleochaetophyceae)

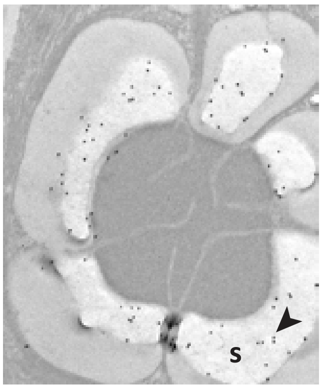

Land plants / Embryophytes

Polytrichum (moss)
(Polytrichopsida)

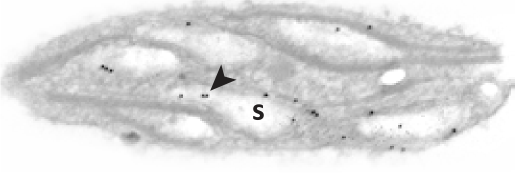

Solanum lycopersicum (tomato)

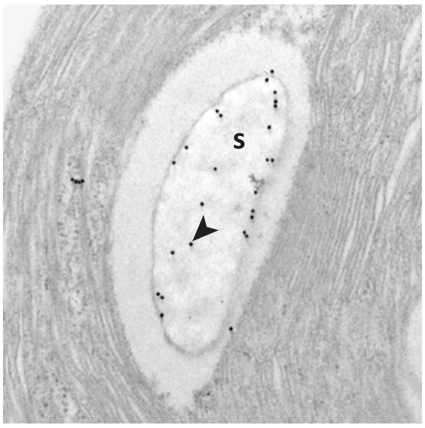

Figure S6. TEM of INCh1
Transmission electron microscopy of diverse Virideplanta. Immunogold labelling (arrowhead) demonstrates the labelling of the starch granules (s).

Figure S7

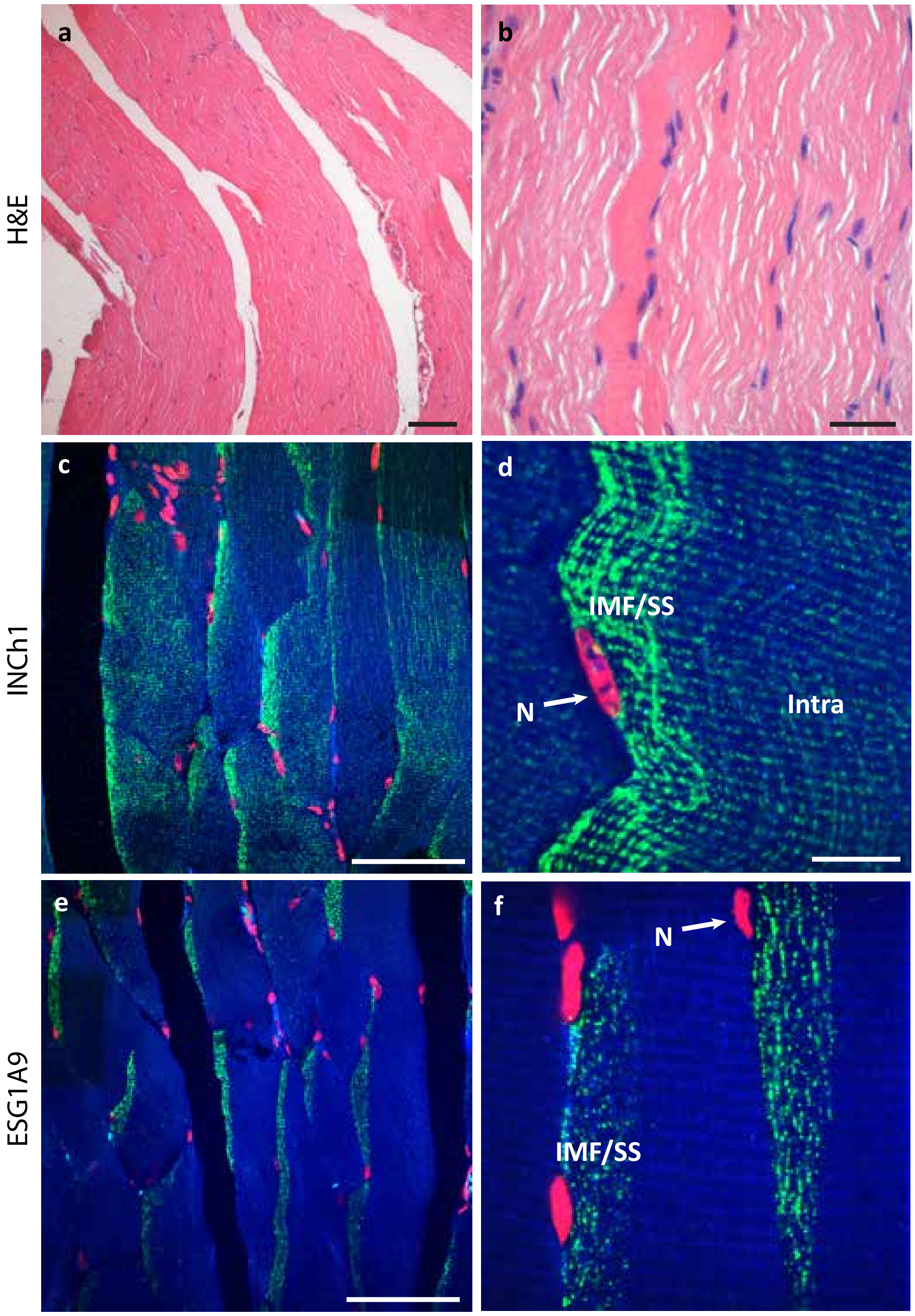

Figure S7. Immunolabelling of glycogen in mouse muscle tissue using INCh1 and ECG1A9
a) Histological analyses of paraffin embedded muscle tissue using hematoxylin and eosin staining. **b)** Higher magnification of S7a. **c)** Immunolabelling of mouse muscle tissue with mAb INCH1 as primary antibody and anti-mouse-IgM conjugated to Alexa Fluor 488 as secondary antibody (green) together with propidium iodide staining of the nulei (red) and UV autofluorescence signal (blue). Note the distribution of INCH1-derived signal throughout the entire muscle tissue suggesting that INCH1 recognizes all forms of glycogen. **d)** Higher magnification of S7c. **e)** Immunolabelling of mouse muscle tissue with mAb ECG1A9 as primary antibody and anti-mouse-IgM conjugated to Alexa Fluor 488 as secondary antibody (green) together with propidium iodide staining of the nulei (red) and UV autofluorescence signal (blue). **f)** High magnification of S7e. Note more restricted localization of the signal when compared to the INCH1 antibody suggesting only inter- and sarcolemal glycogen is labelled. Scale a = 500 µm; b = 100 µm; c and d = 50 µm; d and f = 10µm. Intermyofibrillar glycogen (IMF), intramyofibrilar glycogen (Intra), subsarcolemmal glycogen (SS), nucleus (N).

Figure S8

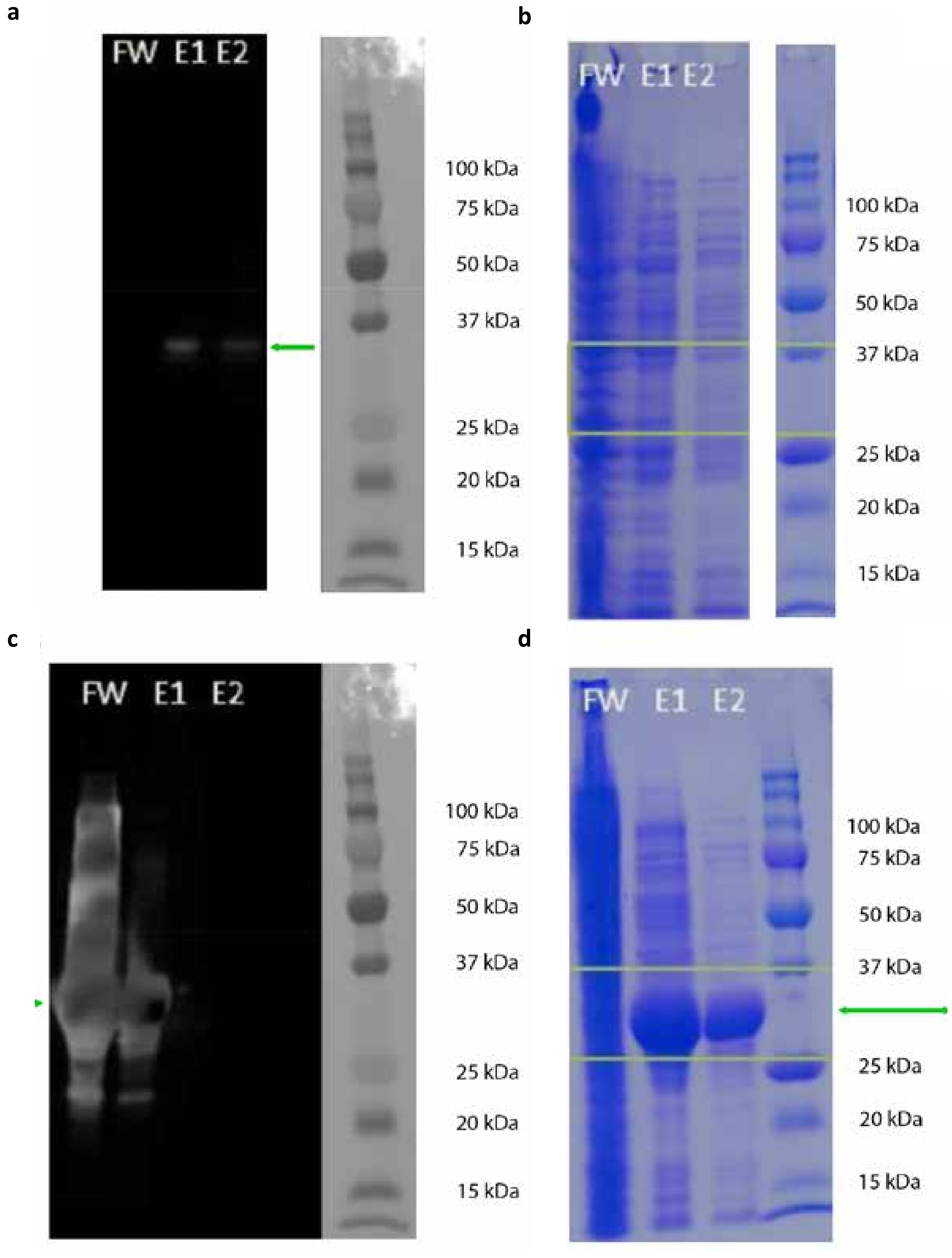

Figure S8. Western blot and SDS PAGE of ulvan lyase
a) western-blot using an anti-His antibody of the ulvan lyase from *Nonlabens ulvanivorans.* **b)** SDS page of ulvan lyase from *Nonlabens ulvanivorans*. **c)** western-blot using an anti-His antibody of the ulvan lyase from *Formosa agariphila*.**d)** SDS page of ulvan lyase from *Formosa agariphila*.

Figure S8

Original scans of all blots in figure S8

Western Blot Anti-His antibody

Image acquisition of the same membranes by

A) chemiluminescence. B) photography

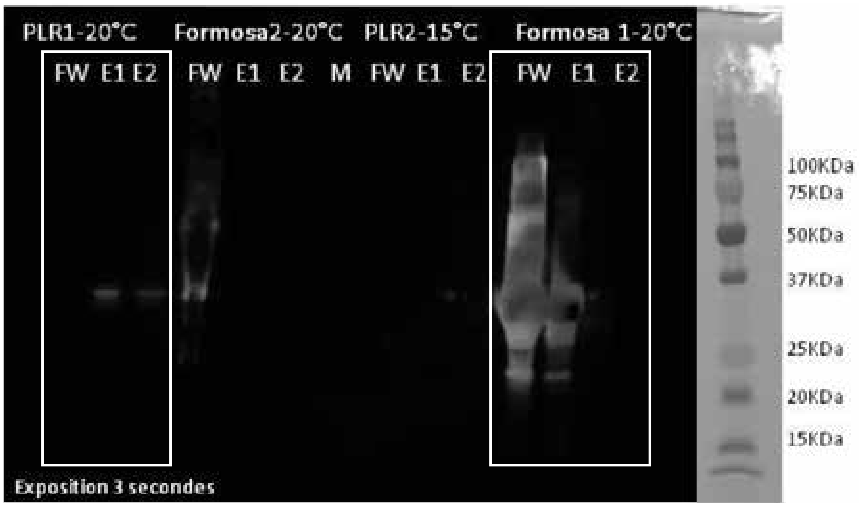

Figure 8a Figure 8c

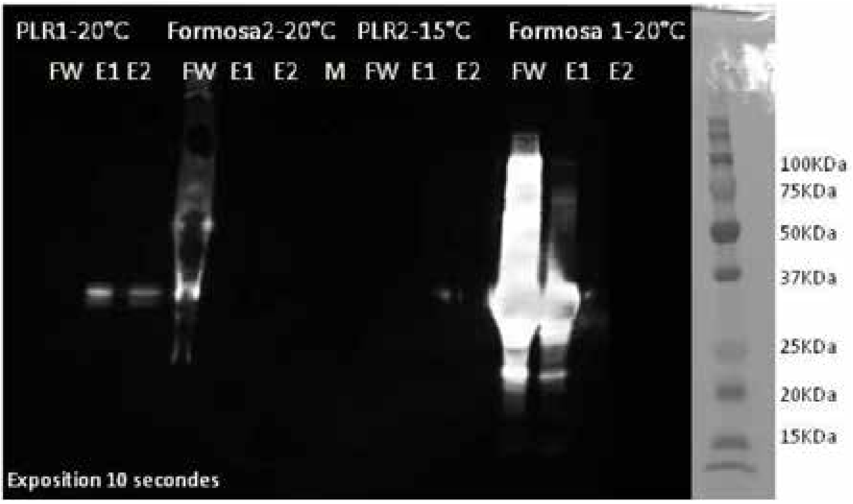

Protein staining of SDS-PAGE

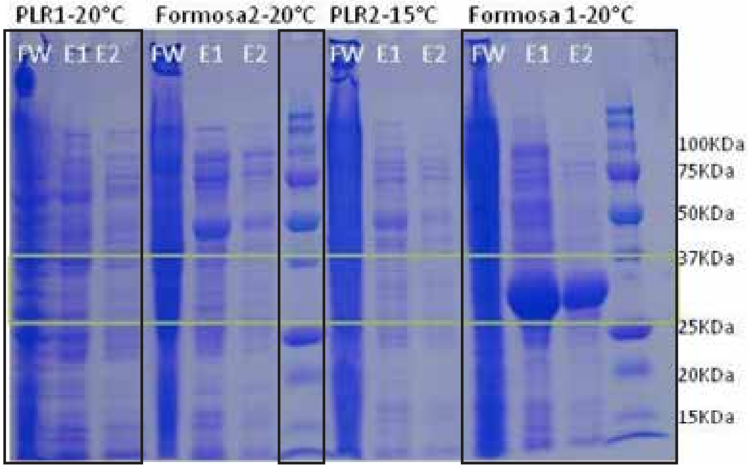

Figure 8b Figure 8b Figure 8d

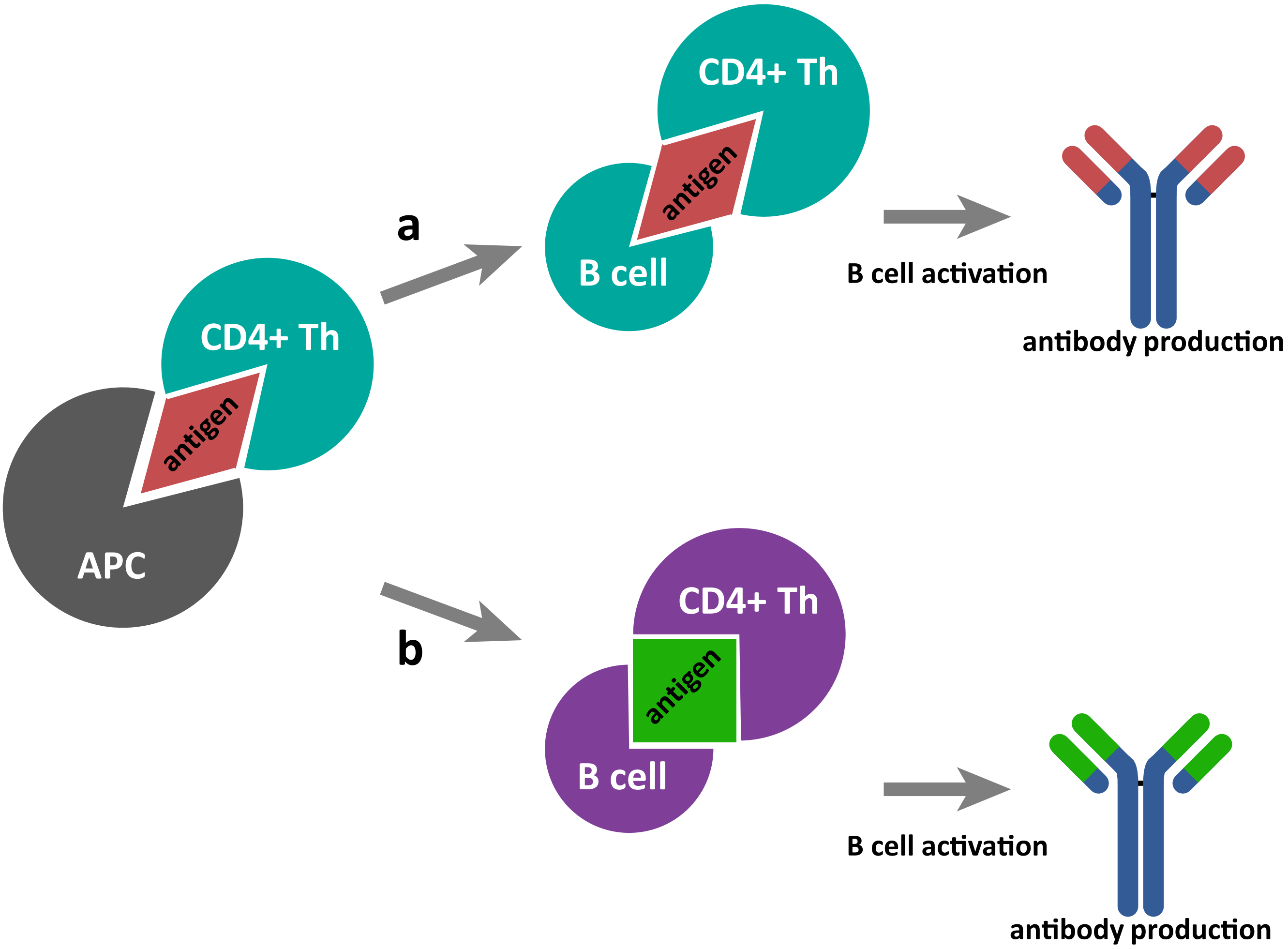

Figure S9. Proposed schematic of immune overload
Scheme of antibody production resulting from immune overload as suggested by current theories. **a)** a normal pathway where the APC binds to the TCR on the CD4+ T-helper cell which then activates the B-cells that bind to that antigen which results in proliferation of those B-cells and production of antibodies against that original antigen. **b)** an immune overload pathway where antigen-presenting cell (APC) binds to the T-cell receptor (TCR) and after TCR-revision – the T-cell activates B-cells that bind to an antigen that is different than the one presented by the APC resulting in activation of B-cells that produce antibodies against different targets.

Figure S10

INCh1

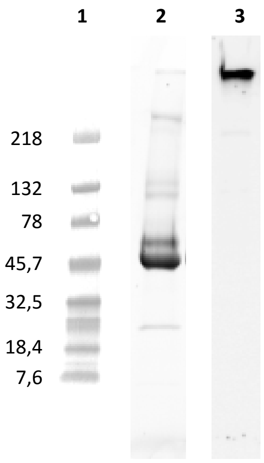

INCh2

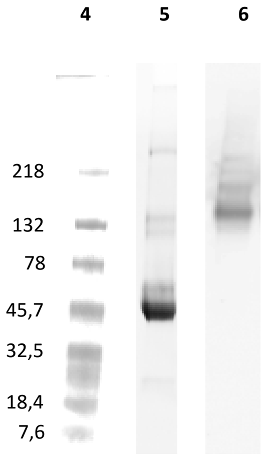

Lane

1: MW standard kDa Biorad (161-0318)
2: INCh1 SDS-PAGE Protein staining
3: INCh1 WB Anti IgM (µ chain) SIGMA A8687

4: MW standard kDa Biorad (161-0318)
5: INCh2 SDS-PAGE Protein staining
6: INCh2 WB Anti IgG (H+L chains) Biorad 170-6516

Figure S10. SDS-PAGE and Western blot analysis of INCh1 and INCh2 showing homogeneity of the respective antibodies.

Figure S10

Original scans of all blots in figure S10

INCh1

Protein staining of SDS-PAGE

Stain Free Image acquisition of the same gel

A) High intensenty B) low intensity

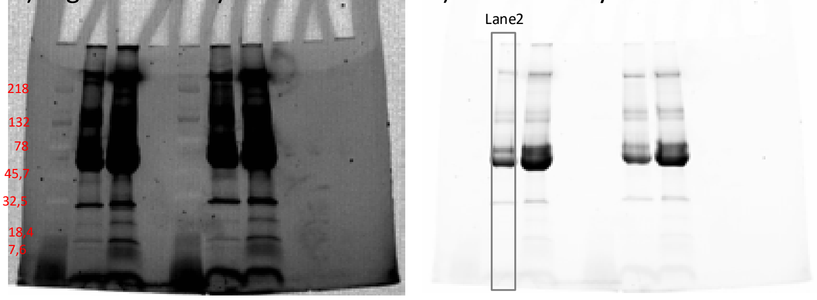

Western Blot Anti IgM

Image acquisition of the same membranes by

A) chemiluminescence. B) photography

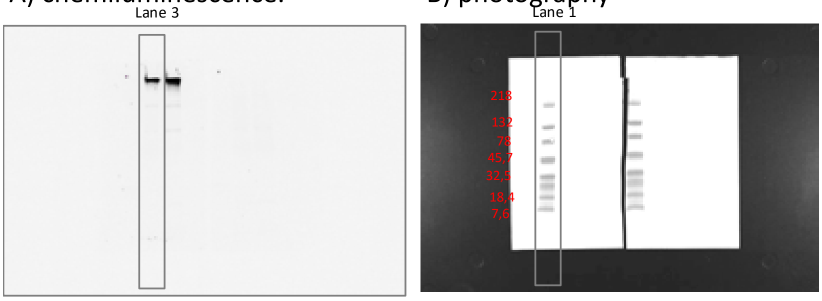

INCh2

Protein staining of SDS-PAGE

Stain Free Image acquisition of the same gel

A) High intensenty B) low intensity

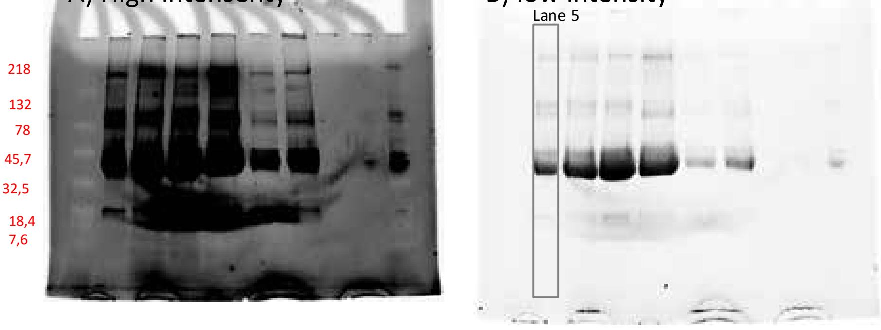

Western Blot Anti IgG

Image acquisition of the same membranes by

A) chemiluminescence. B) photography

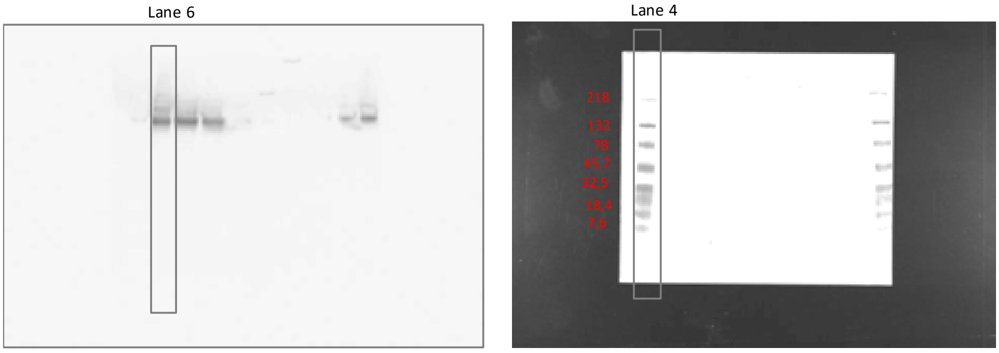

Lane

1: MW standard kDa Biorad (161-0318)
2: INCh1 SDS-PAGE Protein staining
3: INCh1 WB Anti IgM (μ chain) SIGMA A8687

4: MW standard kDa Biorad (161-0318)
5: INCh2 SDS-PAGE Protein staining
6: INCh2 WB Anti IgG (H+L chains) Biorad 170-6516

Table S1

Oligosaccharides

Sample name	Detailed desciption/notes	Source
alginate oligo DP 16.5 (6%M:94%G)	DP 16.5, 94% α(1→4)-L-Guluronic acid, 6% β-(1→4)-D-Mannuronic acid	Dr. Kurt Ingar Draget, Norway
alginate oligo DP 20 (83%M:17%G)	DP 20, 83% β-(1→4)-D-Mannuronic acid, 17% α(1→4)-L-Guluronic acid	Dr. Kurt Ingar Draget, Norway
alginate oligo DP 16 (100%M)	DP 16, 100% β-(1→4)-D-Mannuronic acid	Dr. Kurt Ingar Draget, Norway
fucan oligo DP 2	Oligomer of fucoidan with DP 4, made up of 2 dimeric units of →[3(L-Fuc)α1→4(L-Fuc)α1]→ where the 1,4-substituted fucose unit has sulfate esters in the 2 and 3 position and the 1,3-substituted fucos unit has a sulfate ester in the 2 position	Dr. Gurvan Michel, Roscoff, France
fucan oligo DP 6	Oligomer of fucoidan with DP 6, made up of 3 dimeric units of →[3(L-Fuc)α1→4(L-Fuc)α1]→ where the 1,4-substituted fucose unit has sulfate esters in the 2 and 3 position and the 1,3-substituted fucos unit has a sulfate ester in the 2 position	Dr. Gurvan Michel, Roscoff, France
fucan oligo DP 8	Oligomer of fucoidan with DP 8, made up of 4 dimeric units of →[3(L-Fuc)α1→4(L-Fuc)α1]→ where the 1,4-substituted fucose unit has sulfate esters in the 2 and 3 position and the 1,3-substituted fucos unit has a sulfate ester in the 2 position	Dr. Gurvan Michel, Roscoff, France
agar oligo DP 4	Oligosaccharide from agarose polymer, DP: 4	Dr. Gurvan Michel, Roscoff, France
agar oligo DP 6	Oligosaccharide from agarose polymer, DP: 6	Dr. Gurvan Michel, Roscoff, France
agar oligo DP 8	Oligosaccharide from agarose polymer, DP: 8	Dr. Gurvan Michel, Roscoff, France
agar oligo DP 10	Oligosaccharide from agarose polymer, DP: 10	Dr. Gurvan Michel, Roscoff, France
κ-carrageenan oligo DP 2-24	Sulfated and/or anhydrous galacto oligomer from κ-carrageenan, DP: 2-24	Dr. Gurvan Michel, Roscoff, France
ι-carrageenan oligo	Sulfated and/or anhydrous galacto oligomer, from ι-carrageenan, DP not available	Dr. Gurvan Michel, Roscoff, France
porphyran oligo DP 4	Red algae porphyran Porphyra	Dr. Gurvan Michel, Roscoff, France
porphyran oligo DP 6	Red algae porphyran Porphyra	Dr. Gurvan Michel, Roscoff, France
α-(1-5)-L-arabinobiose (furanose form)	-	Megazyme
α-(1-5)-L-arabinotriose (furanose form)	-	Megazyme
α-(1-5)-L-arabinotetraose (furanose form)	-	Megazyme
α-(1-5)-L-arabinopentaose (furanose form)	-	Megazyme
α-(1-5)-L-arabinoheptaose (furanose form)	-	Megazyme
α-(1-5)-L-arabinooctaose (furanose form)	-	Megazyme
6^1-α-D-galactosyl-β-(1-4)-D-mannobiose	-	Megazyme
6^1-α-D-galactosyl-β-(1-4)-D-mannotriose	-	Megazyme
β-(1-4)-D-mannobiose	-	Megazyme
β-(1-4)-D-mannotriose	-	Megazyme
β-(1-4)-D-mannotetraose	-	Megazyme
β-(1-4)-D-mannopentaose	-	Megazyme
β-(1-4)-D-mannohexaose	-	Megazyme
isoprimeverose	isoprimeverose, α-D-Xylopyranosyl(1→6)-D-Glucose	Megazyme
xyloglucan heptasaccharide, XXXG*	Xyloglucan heptamer, XXXG~OH	Megazyme
XXFG	Xyloglucan oligosaccharide XXFG DP 9 ($C_{51}H_{86}O_{42}$) FW 1371.19	Elicityl
XLFG	Xyloglucan oligosaccharide XLFG DP 10 ($C_{57}H_{96}O_{47}$) FW 1533.33	Elicityl
β-(1-4)-D-xylobiose (pyranose form)	-	Megazyme
β-(1-4)-D-xylotriose (pyranose form)	-	Megazyme
β-(1-4)-D-xylotetraose (pyranose form)	-	Megazyme
β-(1-4)-D-xylopentaose (pyranose form)	-	Megazyme
β-(1-4)-D-xylohexaose (pyranose form)	-	Megazyme
cellotriose	-	Megazyme
cellotetraose	-	Megazyme
cellopentaose	-	Megazyme
cellohexaose	-	Megazyme
β-(1-3)-D-glucosyl-cellobiose	-	Megazyme
β-(1-3)-cellobiosyl-D-glucose	-	Megazyme

Continued

Table S1

β-(1-3)-D-glucosyl-cellotriose	-	Megazyme
β-(1-3)-cellotriosyl-D-glucose	-	Megazyme
β-(1-3)-cellobiosyl-cellobiose	-	Megazyme
laminaribiose	-	Megazyme
laminaritriose	-	Megazyme
laminaritetraose	-	Megazyme
laminaripentaose	-	Megazyme
maltose	-	Sigma-Aldrich
maltotriose	-	Sigma-Aldrich
maltopentaose	-	Sigma-Aldrich
maltohexaose	-	Sigma-Aldrich
maltoheptaose	-	Sigma-Aldrich
α-(1-6)-D-glucosyl-maltotriose	-	Megazyme
6^1-α-maltosyl-maltose	Chemically synthesized	M. S. Motawie
6^3-α-maltosyl-maltotetraose	Chemically synthesized	M. S. Motawie
6^1,6^5-di-α-maltosyl-maltohexaose	Chemically synthesized	M. S. Motawie
6^4-α-maltotriosyl-maltohexaose	Chemically synthesized	M. S. Motawie
6^1-maltose phosphate	Chemically synthesized	M. S. Motawie
3^1-maltose phosphate	Chemically synthesized	M. S. Motawie
N,N'-diacetyl-chitobiose	-	Megazyme
N,N',N''-triacetyl-chitotriose	-	Megazyme
N,N',N'',N'''-tetraacetyl-chitotetraose	-	Megazyme
BSA	Bovine Serum Albumin	Sigma-Aldrich

Polysaccharides

Sample name	Detailed desciption/notes	Source
Alginate (47%M:53%G)	Brown algae, sodium alginate, manuronic/guluronic ratio: 0.9 corresponding to 47% M & 53% G	Danisco (D# 2544-32-11)
Alginate (34%M:66%G)	Brown algae, sodium alginate, manuronic/guluronic ratio: 0.5 corresponding to 34% M & 66% G	Danisco (D# 2544-32-05)
Alginate (25%M:75%G)	Brown algae, sodium alginate, manuronic/guluronic ratio: 0.34 corresponding to 25% M & 75% G	Danisco
Fucoidan	Brown algae, fucoidan, polymer of L-fucose substituted with sulfate esters, can contain up to 20% D-glucoronic acid	Sigma-Aldrich (F5631)
Bacteriological Agar	Bacteriological Agar (agar-agar), no information on content of agarose and agaropectin	Scharlau #07-004-500
Agarose	Agarose, linear polymer made from repeating units of the disaccharide agarobiose, agarobiose contains D-galactose linked β-(1→4) to 3,6-anhydrous L-galactose (reducing end), the agarobiose units are α-(1→3) linked	SeaKem® LONZA LE Agarose
ι-carrageenan	Red algae, iota-carrageenan, repeating dimer: →3[4-SO3--D-Gal]β(1→4)[3,6-anhydro-2-SO3--D-Gal]α(1→	Sigma-Aldrich
λ-carrageenan	Red algae, lambda-carrageenan, repeating dimer: →3[2-SO3--D-Gal]β(1→4)[2,6-diSO3--D-Gal]α(1→	Danisco
κ-carrageenan	Red algae, kappa-carrageenan (D #2544-35-01)	Danisco
Porphyran	Red algae, porphyran polysaccharide. Backbone of 3-linked beta-D-galactosyl units alternating with either 4-linked alpha-L-galactosyl 6-sulfate or 3,6-anhydro-alpha-L-galactosyl units, including 6-O-sulfated L-galactose, 6-O-methylated D-galactose, L-galactose, 3,6-anhydro-L-galactose, 6-O-methyl D-galactose and ester sulfate	Roscoff collaboration
Ulvan (Ulva sp.)	Ulvan polysaccharides from Ulva sp. Native grade, rhamnose 3-sulfate, xylose, xylose 2-sulfate, glucuronic acid and iduronic acide	Elicityl
Ulvan (Enteromorpha sp.)	Ulvan polysaccharides from Enteromorpha sp. Native grade, rhamnose 3-sulfate, xylose, xylose 2-sulfate, glucuronic acid and iduronic acide	Elicityl
Spirulan	spirulan polysaccharide finegrade, acidic sulfated polysaccharide extracted from Spirulina (Arthrospira platensis)	Elicityl

Continued

Table S1

Mannan (Ivory nut)	-	Megazyme
Galactomannan	-	Megazyme
Glucomannan (Konjac)	-	Megazyme
Xylan (Beechwood)	-	Sigma-Aldrich
Arabinoxylan (Wheat)	-	Megazyme
Xyloglucan (Tamarind)	-	Megazyme
Lichenan (Icelandic moss)	-	Megazyme
β-glucan (β-(1-3),(1-6)-glucan) (yeast)	-	Megazyme
β-glucan (β-(1-3), (1-4)-glucan) (oat)	-	Megazyme
β-glucan (β-1,3-glucan) (Euglena gracilis)	-	Megazyme
Pachyman	-	Megazyme
Pullulan	-	Megazyme
Laminarin	-	Sigma-Aldrich
Carboxymethyl cellulose	-	Sigma-Aldrich
Ethyl cellulose	-	Sigma-Aldrich
Methyl cellulose	-	Megazyme
Arabinogalactan (type II)	-	Megazyme
Gum (locust bean)	-	Megazyme
Gum (guar)	-	Sigma-Aldrich
Gum (guaiac)	-	Sigma-Aldrich
Gum (karaya)	-	Sigma-Aldrich
Gum (ghatti)	-	Sigma-Aldrich
Gum (arabic)	-	Sigma-Aldrich
Lime pectin DE: 81% (E81)*	-	Danisco
Lime pectin DE: 15% (B15)*	-	Danisco
Lime pectin DE: 34% (B34)*	-	Danisco
Lime pectin DE: 43% (B43)*	-	Danisco
Lime pectin DE: 64% (B64)*	-	Danisco
Lime pectin DE: 71% (B71)*	-	Danisco
Lime pectin DE: 11% (F11)*	-	Danisco
Lime pectin DE: 43% (F43)*	-	Danisco
Lime pectin DE: 76% (F76)*	-	Danisco
Lime pectin DE: 16% (P16)*	-	Danisco
Lime pectin DE: 53% (P53)*	-	Danisco
Lime pectin DE: 76% (P76)*	-	Danisco
Pectin (sugar beet)	-	Danisco
Linear arabinan	-	Megazyme
Pectic galactan (lupin)	-	Megazyme
Rhamnogalacturonan (soy bean)	-	Megazyme
Rhamnogalacturonan I (potato)	-	Megazyme
Rhamnogalacturonan I (Citrus)	-	M. C. Ralet
Polygalacturonic acid	-	Megazyme
Pectin (lemon)	-	J. P. Knox
Pectin (apple)	-	J. P. Knox
Galactan (potato)	-	Megazyme
Feruloylated pectin (sugar beet)	-	M. C. Ralet
Amylose (potato)	-	Sigma-Aldrich
Amylopectin (potato)	-	Sigma-Aldrich
Glycogen (mussel)	-	A. Blennow
Starch (potato)	-	Andelskartoffelmelsfabrikken, Denmark
Starch (maize)	-	Cerestar-AKV, Iceland
Starch (waxy maize)	-	A. Blennow
Starch (tapioca)	-	A. Blennow
Starch (wheat)	-	A. Blennow
Starch (pea)	-	Kartoffelmelcentralen, Denmark
Dextran	-	A. Blennow
Starch (Brachipodium)	-	V. Tanackovic
Floridian starch (red algae)	-	S.Koutaniemi
Chondroitin sulfate A	chondroitin sulfate A (animal, sulfated, uronic acids)	Sigma-Aldrich
Chondroitin sulfate B	chondroitin sulfate B (animal, sulfated, uronic acids)	Sigma-Aldrich
Heparan sulfate	heparan sulfate (animal, sulfated, uronic acids)	Sigma-Aldrich
Hyaluronic acid	hyaluronic acid (animal, uronic acid)	Sigma-Aldrich

* p series — blockwise de-esterification of E81 with a pPME isolated from orange peel (P-series)
f series — non-blockwise de-esterification of E81 with a fPME from Aspergillus niger
b series — non-blockwise de-esterification of E81 by base catalysis

In accordance with Willats et al. 2001 (‘Modulation of the Degree and Pattern of Methyl-esterification of Pectic Homogalacturonan in Plant Cell Walls: IMPLICATIONS FOR PECTIN METHYL ESTERASE ACTION, MATRIX PROPERTIES, AND CELL ADHESION’, J. Biol. Chem. 2001, 276:19404-19413. doi: 10.1074/jbc.M011242200 originally published online March 6, 2001)

Table S2

Algae species in immunogen with growth conditions and growth media

Phylum	Class	Order	Family	Genus	Species	Growth medium	Growth temp/light
Charophyta	Charophyceae	Charales	Characeae	Chara	corallina	Aquarium/pond water	Natural light
Charophyta	Chlorokybophyceae	Chlorokybales	Chlorokybaceae	Chlorokybus	atmophyticus	WH2XS	A
Charophyta	Coleochaetophyceae	Coleochaetales	Coleochaetaceae	Coleochaete	nitellarum	WH2XS	A
Charophyta	Coleochaetophyceae	Coleochaetales	Coleochaetaceae	Coleochaete	orbicularis	3NBBM	A
Charophyta	Zygnematophyceae	Desmidiales	Closteriaceae	Closterium	acerosum	WH2XS	A
Charophyta	Zygnematophyceae	Desmidiales	Desmidiaceae	Micrasterias	furcata	WH2XS	A
Charophyta	Zygnematophyceae	Desmidiales	Desmidiaceae	Teilingia	granulata	WH2XS	A
Charophyta	Zygnematophyceae	Desmidiales	Desmidiaceae	Tetmemorus	sp.	WH2XS	A
Charophyta	Zygnematophyceae	Desmidiales	Desmidiaceae	Cosmarium	turpini	Waris	A
Charophyta	Zygnematophyceae	Zygnematales	Mesotaeniaceae	Cylindrocystis	sp.	WH2XS	A
Charophyta	Zygnematophyceae	Zygnematales	Mesotaeniaceae	Mesotaenium	caldariorum 41	3NBBM	A
Charophyta	Zygnematophyceae	Zygnematales	Mesotaeniaceae	Netrium	interruptum (335)	WH2XS	A
Charophyta	Zygnematophyceae	Desmidiales	Peniaceae	Penium	margaritaceum	WHS	A
Charophyta	Zygnematophyceae	Zygnematales	Zygnemataceae	Mougeotia	transeau	3NBBM	A
Charophyta	Zygnematophyceae	Zygnematales	Zygnemataceae	Spirogyra	communis	3NBBM	A
Charophyta	Zygnematophyceae	Desmidiales	Desmidiaceae	Pleurotaenium	trabecula	WH2XS	A
Charophyta	Klebsormidiophyceae	Klebsormidiales	Klebsormidiaceae	Klebsormidium	flaccidum 323	3NBBM	A
Charophyta	Klebsormidiophyceae	Klebsormidiales	Klebsormidiaceae	Klebsormidium	321	WH2XS	A
Charophyta	Klebsormidiophyceae	Klebsormidiales	Klebsormidiaceae	Klebsormidium	dissectum 2155	WH2XS	A
Charophyta	Klebsormidiophyceae	Klebsormidiales	Klebsormidiaceae	Entransia	2353	WH2XS	A

Phylum	Class	Order	Family	Genus	Species	Growth medium	Growth temp/light
Prasinophyta	Pedinophyceae	Pedinomonadales	Pedinomonadaceae	Pedinomonas	minor	WHS2XS	A
Prasinophyta	Prasinophyceae	Mamiellales	Dolichomastigaceae	Dolichomastix	tenuilepis	F/2	A

Continued

Table S2

Phylum	Class	Order	Family	Genus	Species	Growth medium	Growth temp/light
Chlorophyta	Chlorodendrophyceae	Chlorodendrales	Chlorodendraceae	Tetraselmis	striata	AlgaGrow-Sea	A
Chlorophyta	Chlorophyceae	Oedogoniales	Oedogoniaceae	Oedogonium	foveolatum	3NBBM	A
Chlorophyta	Chlorophyceae	Chlamydomonadales	Chlamydomonadaceae	Chlamydomonas	reinhardii	3NBBM	A
Chlorophyta	Chlorophyceae	Chlamydomonadales	Haematococcaceae	Haematococcus	pluvialis	WH2XS	A
Chlorophyta	Chlorophyceae	Sphaeropleales	Scenedesmaceae	Scenedesmus	quadricauda	WHS	A
Chlorophyta	Chlorophyceae	Chlamydomonadales	Volvocaceae	Pandorina	morum	WHS	A
Chlorophyta	Chlorophyceae	Sphaeropleales	Hydrodictyaceae	Hydrodictyon	sp.	BBM	A
Chlorophyta	Chlorophyceae	Chlamydomonadales	Dunaliellaceae	Dunaliella	tertiolecta	AlgaGrow-Sea	A
Chlorophyta	Pyramimonadophyceae	Pseudoscourfieldiales	Pycnococcaceae	Pseudoscourfieldia	marina	Erd-Schreiber	A
Chlorophyta	Pyramimonadophyceae	Pyramimonadales	Pyramimonadaceae	Pyramimonas	parkeae	AlgaGrow-Sea	A
Chlorophyta	Trebouxiophyceae	Chlorellales	Chlorellaceae	Chlorella	sp.	WHS	A
Chlorophyta	Trebouxiophyceae	Chlorellales	Chlorellaceae	Pseudochloris	sp.	F/2	A
Chlorophyta	Ulvophyceae	Cladophorales	Cladophoraceae	Cladophora	glomerata	Natural collection	Natural light
Chlorophyta	Ulvophyceae	Ulvales	Ulvaceae	Enteromorpha	intestinalis 3170	Alga Grow-Sea	A
Chlorophyta	Ulvophyceae	Ulotrichales	Ulotrichaceae	Interfilum	2167	WH2S	A
Chlorophyta	Ulvophyceae	Ulvales	Ulvaceae	Ulva	lactuta	AlgaGrow-Sea	A
Chlorophyta	Ulvophyceae	Bryopsidales	Codiaceae	Codium	sp.	Natural collection	Natural light
Chlorophyta	Ulvophyceae	Oltmannsiellopsidales	Oltmannsiellopsidaceae	Oltmannsiellopsis	unicellularis	K medium	A
Chlorophyta	Nephroselmidophyceae	Nephroselmidales	Nephroselmidaceae	Nephroselmis	pyriformis	F/2	A
Chlorophyta	Ulvophyceae	Ulotrichales	Ulotrichaceae	Ulotrix	fimbrata	WHS	A

A: 18°C ± 1°C, 14:10 light/darkness regime, 35 W/m2 of cool white fluorescent light

Growth medium

Name	Source
F/2	https://ncma.bigelow.org/media/wysiwyg/Algal_recipes/NCMA_algal_medium_f_2_1.pdf
BBM	http://cccryo.fraunhofer.de/sources/files/medien/BBM.pdf
3NBBM	http://www.ccap.ac.uk/media/documents/MBBM.pdf
Alga-Gro Seawater	Purchased from Carolina Biological Supply Company
Erd-Schreiber	https://www.mba.ac.uk/erd-schreiber-erds-culture-medium/
K medium	https://ncma.bigelow.org/media/wysiwyg/Algal_recipes/NCMA_algal_medium_K_1.pdf
Waris	http://web.biosci.utexas.edu/utex/Media%20PDF/waris%20medium.pdf
WH	http://www.ccap.ac.uk/media/documents/MWC.pdf
WHS and WH2XS	Woods Hole medium with 5% soil extract (Carolina Biological)

Table S3

Assesment of secondary tagged anti-mouse IgG with IgM antibodies

	**Biorad**<br><br>**anti IgG (H+L chains)**	**SIGMA (A8687)**<br><br>**anti IgM (µ chain)**
**20.000**	2,238	1,671
**5.000**	1,804	1,547
**1.250**	1,144	1,151
**313**	0,482	0,848
**78**	0,168	0,409
**20**	0,054	0,152
**5**	0,024	0,054
**1**	0,015	0,018
**20.000**	2,238	1,671
**5.000**	1,804	1,547

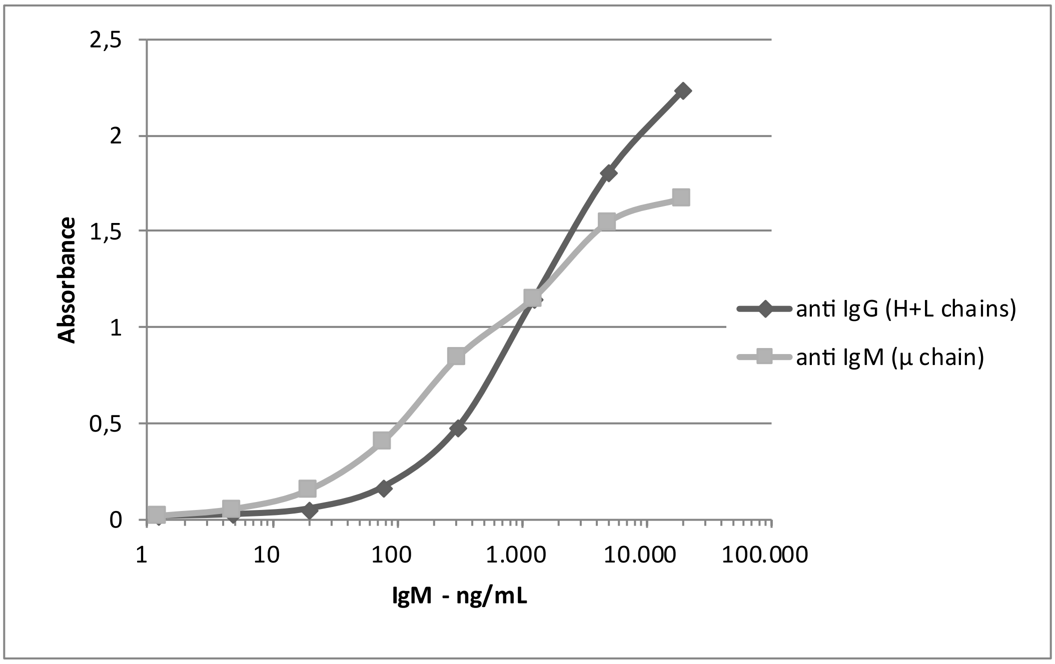

Table S4

Enzymes used for epitope deletion

Enzyme	Product code	pH	buffer	Temp
Pullulanase M1 (*Klebsiella planticola*)	E-PULKP	5	0.2M Na Acetate buffer	40
Isoamylase (*Pseudomonas sp.*)	E-ISAMY	4,5	0,5 M Na Acetate buffer	40
Amyloglucosidase (*Aspergillus niger*)	E-AMGDF	5	0.2M Na Acetate	40
α-Amylase (*Aspergillus oryzae*)	E-ANAAM	7	200 mM Na Phosphate buffer	50
Endo-ß-1,3-D-Glucanase (*Trichoderma sp.*)	E-LAMSE	4,5	0,5 M Na Acetate buffer	40
Endo-ß-1,4-Mannanase (*Cellvibrio japonicus*)	E-BMACJ	7,0	200 mM Na Phosphate buffer	50
Pectate lyase (*C. japonicus*)	E-PLYCJ	7,0	200 mM Na Phosphate buffer	40
Endo-cellulase (EGII) (*Trichoderma longibrachiatum*)	E-CELTR	4,5	0,5 M Na Acetate buffer	40
Endo-ß-1,4-Xylanase M4 (*Aspergillus niger*)	E-XYAN4	5	0.2M Na Acetate	40
Ulvan lyase (*Formosa agariphila*)	-	7,5	100 mM MOPS, 50 mM NaCl	RT
